# Molecular Mechanisms of Neuroinflammation in Aging and Alzheimer’s Disease Progression

**DOI:** 10.3390/ijms24031869

**Published:** 2023-01-18

**Authors:** Felicia Liana Andronie-Cioara, Adriana Ioana Ardelean, Carmen Delia Nistor-Cseppento, Anamaria Jurcau, Maria Carolina Jurcau, Nicoleta Pascalau, Florin Marcu

**Affiliations:** 1 Department of Psycho-Neurosciences and Rehabilitation, Faculty of Medicine and Pharmacy, University of Oradea, 410073 Oradea, Romania; 2 Department of Preclinical Sciences, Faculty of Medicine and Pharmacy, University of Oradea, 410073 Oradea, Romania; 3 Faculty of Medicine and Pharmacy, University of Oradea, 410073 Oradea, Romania

**Keywords:** neuroinflammation, inflammaging, Alzheimer’s disease, microglia, cellular senescence, TNF signaling, TREM2, oxidative stress, therapy

## Abstract

Aging is the most prominent risk factor for late-onset Alzheimer’s disease. Aging associates with a chronic inflammatory state both in the periphery and in the central nervous system, the evidence thereof and the mechanisms leading to chronic neuroinflammation being discussed. Nonetheless, neuroinflammation is significantly enhanced by the accumulation of amyloid beta and accelerates the progression of Alzheimer’s disease through various pathways discussed in the present review. Decades of clinical trials targeting the 2 abnormal proteins in Alzheimer’s disease, amyloid beta and tau, led to many failures. As such, targeting neuroinflammation via different strategies could prove a valuable therapeutic strategy, although much research is still needed to identify the appropriate time window. Active research focusing on identifying early biomarkers could help translating these novel strategies from bench to bedside.

## 1. Introduction

Medical achievements and public health efforts over the last decades have contributed to reducing mortality in early and midlife from infectious and cardiovascular diseases as well as from some forms of cancer, increasing global life expectancy from 66.20 years in 1995 to 72.98 years in 2017, the top 5 countries being Singapore (84.79 years), Japan, Switzerland, Italy, and Kuwait [1]. However, because aging is the major risk factor for neurodegenerative diseases, these increasing numbers of aged persons now face the risk of developing such diseases simply due to their survival. Among neurodegenerative diseases, Alzheimer’s disease is the most common form of dementia, affecting currently about 50 million people worldwide, with numbers expected to triple by 2050 [2]. Aside from the heavy burden on healthcare and social systems as well as on families posed by Alzheimer’s disease (AD), it also robs affected individuals of the attributes that make long lives worth living, such as memories, feelings, thinking, or the ability to make decisions [3].

Despite the ambition set by the G8 dementia summit held in 2013 to identify a disease-modifying treatment for AD by 2025 [4], decades of clinical trials driven by the amyloid cascade hypothesis have failed. Meanwhile, research has provided compelling evidence on the involvement of neuroinflammation in both aging and AD pathogenesis, identifying new targets that could help us stop the inexorable progression of this devastating disease.

## 2. Aging and Immunity

Aging is accompanied by an impairment of the function of the immune system affecting both the innate and adaptive responses; raising the susceptibility of elderly patients to bacterial, viral and fungal infections; and diminishing their immune response to vaccines [5]. The phagocytic activity of neutrophils and macrophages, as well as that of natural killer lymphocytes, is reduced [6]. In the adaptive immune system, one of the major changes is the involution of the thymus, leading to the reduced production of new and naïve T cells and an increase in terminally differentiated memory T cells [7]. In adults, the naïve T cell pool is also maintained through proliferation, but aged naïve T cells show defective expansion [8]. In addition, autoreactive T cell clones, no longer depleted by the thymus, may be released into the periphery and increase susceptibility to autoimmune reactions [9]. The number of circulating B cells also diminishes, and B lymphocytes exhibit a reduced antibody repertoire [10], more prone to reacting against self-antigens. All these dysregulations are accompanied by increased circulating markers of inflammation, leading to chronic low-grade inflammation, known as inflammaging [11].

### 2.1. Neuroinflammation in the Aging Brain

The central nervous system (CNS) has for long been considered an immune-privileged site due to a lack of rapid adaptive immune response to foreign antigens [12]. Nonetheless, research has shown that the CNS does communicate with the immune system [13].

The resident innate immune cells of the CNS are microglia. During development, primitive myeloid precursors arise from the yolk sac following the expression of runt-related transcription factor 1 (RUNX1) and macrophage colony stimulating factor 1 receptor (CSF1R), after which they reach the embryonic head through the blood flow and migrate to the developing brain by using matrix metalloproteinases [14]. Following migration, the maintenance of the population relies on the self-renewal of microglia [14].

Resting microglia have commonly fixed somata with motile filopodia-like processes with different morphologies that enable the cells to carry out immune surveillance throughout the parenchyma [15]. Neuronal signals maintain the resting state in the microglia, characterized by a low expression of CD68, through the expression of a distinct set of proteins, including CX_3_CL1, CD22, the neuronal plasma membrane marker CD200, neurotransmitters, and neurotrophins, which interact with corresponding receptors on microglial cells [16]. In addition to the low expression of CD68, resting microglia downregulate MHC-I and MHC-II, as opposed to activated microglia, that express high levels of MCH-II and co-stimulatory antigens. This enables microglia to interact with antigen-presenting cells and present antigens to T-lymphocytes [17].

While constantly surveying the environment, microglia recognize bacterial or viral molecules as well as endogenous proteins and DNA or RNA released by damaged cells via runt-related transcription factor 1 (belonging to the PRRs) located mainly on the microglial plasma membrane. These PRRs are mostly toll-like receptors (TLRs), triggering receptor expressed on myeloid cells (TREMs) or nucleotide-binding oligomerization domain (NOD)-like receptors (NLRs) [18]. The interaction of the various ligands with these receptors triggers a series of signaling pathways that lead to an upregulated CD68 profile and the production of pro-inflammatory cytokines, such as interleukin (IL)-1β, IL-6, IL-18, tumor necrosis factor (TNF)-α, and cyclooxygenase-2 (COX2), of chemokines such as C-C motif chemokine ligand 1 (CCL1), CCL5, and C-X-C motif ligand 1 (CXCL1), of small-molecule messengers (prostaglandins, nitric oxide, reactive oxygen species), and interferons to mediate the neuroinflammatory response [19,20]. In the frame of this pro-inflammatory response, the microglial phagocytosis of damaged cells and neurotoxic aggregates is promoted [18]. In terms of cellular morphology, activated microglia take on an amoeboid shape (“puff up”) to enable the phagocytosis of foreign or damaged cells or proteins. To achieve an efficient response, microglia cooperate with astrocytes, capillary endothelial cells, as well as infiltrating blood cells that can gain access through a “leaky” blood–brain barrier (BBB) [19]. Injury-related ATP release can induce an astrocyte-derived ATP gradient that is sensed by microglia through the purinergic receptor P2RY12 and leads to rapid microglial migration and activation [21]. In addition, IL-1α, TNF-α, and C1q, secreted by activated microglia, are able to induce a so-called ‘A1′ or neurotoxic reactive astrocyte phenotype [22]. Systemic inflammation causes endothelial cells to release chemokines, such as CCL5, which triggers microglial cells to express CLDN5 and to infiltrate through the neurovascular unit contacting endothelial cells and forming tight junctions to maintain the integrity of the BBB. However, sustained inflammation causes microglia to polarize toward the phagocytic phenotype, engulfing astrocytic fragments and end feet and weakening the BBB [23]. This creates the premises for cells of the adaptive immune response, such as macrophages and lymphocytes, to infiltrate and interact with cells of the innate immune system via chemokines and their receptors. For example, CCR5 promotes neuroinflammation, while CCR2 shifts microglial polarization toward the anti-inflammatory M2 phenotype. In addition to chemokines and their receptor signaling, cytokines released by infiltrating cells may additionally modulate the immune response [24]. Anti-inflammatory cytokines, such as IL-1 receptor antagonist, IL-4, IL-10, IL-11, prevent excessive inflammation and favor the shifting of the microglia toward an anti-inflammatory M2 phenotype, promoting tissue repair [25]. Although the classical dichotomy of M1 (pro-inflammatory) and M2 (anti-inflammatory) microglia is probably a simplified version of the story, it is still used by researchers to convey the idea that microglia can be both detrimental and protective [26]. Nonetheless, recent research has revealed significant variations in the transcription signature of activated microglia accompanying various brain insults, such as trauma, infection, or neurodegeneration [15].

In the aging brain, microglia as well as glial cells have a series of functional impairments that contribute to sustained activation, maintaining the chronic neuroinflammatory state [27], together with immunosenescence [11], mitochondrial dysfunction [28], impaired mitophagy and autophagy [29,30], dysfunction of the ubiquitin–proteasome system [31], meta-inflammation caused by obesity [32], and gut dysbiosis [33]. All these impairments and abnormalities lead to increases in the circulating C reactive protein, IL-6 [34,35], TNF-α and its receptors [36], intercellular adhesion molecule 1 (ICAM-1), tissue inhibitor of metalloproteinases 1 (TIMP-1), the astrocytic intermediate filament glial fibrillary acidic protein (GFAP), and markers of mitochondrial dysfunction, such as growth/differentiation factor 15 (GDF15) and fibroblast growth factor 21 (FGF21) [37], translated clinically into physical frailty and sarcopenia [37]. The neuroimmune interactions between the CNS and the periphery are bidirectional, IL-6, for example, being able to cross the BBB or gain access to the CNS through regions with increased permeability to the circulating milieu such as the circumventricular organs [38].

#### 2.1.1. Mitochondria in the Aging Brain

Specifically for the brain, the high energy demands used mainly for synaptic transmission, synaptogenesis, and synaptic pruning are supplied mainly by mitochondria via oxidative phosphorylation (OXPHOS) [39], while astrocytes are mainly glycolytic and provide lactate and other small molecules to neurons to be oxidized by neuronal mitochondria and provide ATP [40]. Although up to 11 distinct sites where ROS can be produced have been identified in isolated mitochondria [41], only respiratory complex I (ubiquinone oxidoreductase), complex II (succinate dehydrogenase), and complex III (cytochrome *c* reductase) are relevant for ROS production in vivo [42]. Even under normal conditions, about 2% of electrons “leak” from the electron transport chain (ETC) and will generate reactive oxygen species (ROS). ROS produced by complex I are directed to the mitochondrial matrix and are involved in cellular differentiation and damage caused by ischemia/reperfusion injuries [43], while ROS produced by complex III are divided between the matrix and intermembrane space and trigger the hypoxia response [44]. Respiratory complexes are organized into supercomplexes, which vary in response to changes in substrate availability [45]. Other sources of mitochondrial superoxide include α-ketoglutarate dehydrogenase, pyruvate dehydrogenase, glycerol-3-phosphate dehydrogenase, and fatty acid beta-oxidation [46]. Neurons, having less antioxidant defense systems, are more sensitive than glia to oxidative damage [42]. Moreover, specific subpopulations of neurons, mainly large neurons with long axons, show selective vulnerability to oxidative stress [47]. These neurons are located mainly in the frontal cortex, amygdala, substantia nigra, or hippocampus [42].

Aging associates changes in the structure and function of mitochondria, presumably related to oxidative stress. Mitochondrial age-related changes consist of excessive fragmentation (in the CA1 region of the hippocampus) [48] or enlargement (for example in the frontal cortex) [49]. The activity of complex I of the respiratory chain [50] and complex IV decreases [42], with important consequences on the rate of ROS production. The reduction of complex IV activity increases the redox state of the ETC, stimulating electron leakage and the production of ROS [42]. Whether the accumulation of damaged mitochondria is the result of ROS attack (the traditional view) or, on the contrary, the accumulation of mitochondrial ROS results from reduced mitochondrial activity, as suggested by a mouse model with a knock-in mutation of POLG (DNA polymerase subunit gamma, in charge of replicating and repairing mitochondrial DNA), which shows a significant reduction of mitochondrial respiration without accumulating excess ROS levels [51], is still a matter of debate.

The physical proximity of the generated ROS with mitochondrial proteins makes the latter highly susceptible to oxidative damage, further decreasing the energetic efficiency of aged mitochondria and impairing the ATP-requiring fast axonal transport of the organelles to sites of high energetic demand, usually the synaptic sites [52]. The mitochondria-generated ROS also damage mitochondrial DNA (mtDNA), which is about ten times more prone to oxidative attack compared to nuclear DNA because it lacks protective histones [53]. Although endowed with repair mechanisms such as the base excision repair mechanism (BER) or a specific version of DNA ligase III that participates in DNA replication and repair, and in spite of the contribution of mitochondrial fusion in safeguarding the mitochondrial genome integrity, mtDNA damage may accumulate over years and can propagate through clonal expansion, exacerbating neurodegeneration [54,55,56].

Alterations in ROS signaling impair the mechanisms of quality control, making cells more vulnerable to senescence [57] and cell death [58]. The decline in the respiratory function and alterations of the function of transcription factors also alters neural biogenesis [59,60].

Another important function of mitochondria is the buffering of excess cytosolic calcium [30]. Although neurons in different brain regions have different protein expression patterns and different content of calcium-binding proteins [61], the changes in mitochondrial morphology and function alter also their Ca^2+^-buffering capacity. Ca^2+^ entry through the outer mitochondrial membrane is mediated by voltage-dependent anion channel 1 (VDAC1), while the transport from the intermembrane space to the matrix is mediated by the membrane Ca^2+^ uniporter (MCU) scaffolded by essential MCU regulator (EMRE), mitochondrial calcium uptake 1 and 2 (MICU1, MICU2), and MCU regulator 1 (MCUR1) [62]. Calcium efflux is mediated by the Li^+^-permeable Na^+^/Ca^2+^ exchanger (NCLX) [39]. In buffering Ca^2+^, mitochondria cooperate with the endoplasmic reticulum (ER) through mitochondria-associated membranes (MAMs), the core of which contains inositol trisphosphate receptors (IP3Rs) that interact with the mitochondrial chaperone glucose-regulated protein 75 (Grp75) and the VDACs [63]. By damaging VDACs, ROS contribute to the impairment in mitochondrial calcium buffering, which, together with the age-dependent enhanced expression of the N-type voltage-gated calcium channel (VGCC) [64] and the increased activity of L- and N-type VGCCs, leads to increased cytosolic calcium levels with the subsequent formation of the mitochondrial permeability transition pore (MPTP) and apoptosis [62].

Although glial cells have been less extensively studied, research has revealed that they may even outweigh the importance of neurons in the process of aging [65]. Age- and ROS-induced genomic alterations lead to mitochondrial dysfunction and apoptosis in glial cells as well, while ROS are able to induce a permanent cell-cycle arrest known as cellular senescence, affecting both mitotic cells, such as glia and postmitotic neurons. The senescent phenotype is characterized by the secretion of pro-inflammatory factors that can, in turn, induce senescence in neighboring cells [66].

Due to the “endosymbiont” nature of mitochondria, thought to originate from an α-proteobacteria that entered a symbiotic relationship with an ancestral eukaryotic cell [67], the organelle has circular double-stranded mitochondrial DNA containing cytosine phosphate guanosine, cardiolipin, and N-formylated peptides, which are all bacterial features recognized by immune cells. Once released via the opening of the MPTP and the loss of cellular membrane integrity, these molecules are recognized as damage-associated molecular patterns (DAMPs) or pathogen-associated molecular patterns (PAMPs) and interact with pattern recognition receptors (PRRs) present on microglia, astrocytes, and macrophages. As such, they prime the process of antigen presentation by dendritic cells and lead to the expression of proinflammatory molecules and nitric oxide (NO) in microglial cells of the CNS [68]. Among these, DAMPs are also cytochrome c, cardiolipin, and mitochondrial transcription factor A (TFAM). Once in the extracellular space, cytochrome c increases the secretion of nitric oxide and the production of ROS, likely by binding to toll-like receptor 4 (TLR4) [69], followed by the activation of the Jun N-terminal kinase (JNK) pathway [70], the production of reactive nitrogen species, and the activation of signaling cascades downstream of PRRs, such as the mitogen-activated protein kinase (MAPK) signaling cascades. Externalized cardiolipin upregulates the phagocytic activity of immune cells [71] and modulates the release of cytokines [72]. TFAM is a member of the high-mobility box group (HMBG) proteins localized under physiological conditions in the IMM [68]. Released into the extracellular space following cellular damage, it possibly forms complexes with interferon 1γ [73] and acts as a pro-inflammatory signaling molecule, activating microglia and increasing the production of IL-1β, IL-6, IL-8, and TNF-α [73].

#### 2.1.2. Oxidative Stress and Brain Aging

A landmark study comparing the levels of oxidized nucleoside 8-hydroxy-2′-deoxyguanosine (OH8dG) in nuclear and mitochondrial DNA isolated from various regions of the cerebral cortex and cerebellum of humans aged 42 to 97 years found a progressive increase of OH8dG with age, although the increase was significantly more pronounced in mtDNA [74]. A subsequent study comparing the markers of DNA damage in patients with AD found a 3-fold increase in mtDNA oxidative damage compared to nuclear DNA [75]. However, the increased levels of unrepaired double-strand breaks and the age-related downregulation of the expression of the DNAse TREX1 (three prime repair exonuclease 1) leads to increased levels of cytoplasmic DNA [76].

The composition of phospholipids in aged human brains is altered as well, with increased markers of lipid peroxidation (malondialdehyde levels) and intraneuronal lipofuscin deposits being identified in aged brain samples [77]. Likewise, protein oxidation markers (carbonyl residues) are increased as well [78].

Mitochondrial membrane fluidity (caused by lipid peroxidation) is altered in aged individuals and AD patients. Specifically in AD patients, the mitochondrial membrane fluidity was maximally altered and could not be further impaired by exposing mitochondria to peroxidizing conditions [79]. The activity of certain enzymes, such as aconitase 2 (an enzyme of the Krebs cycle), was found to be reduced in the lymphocytic mitochondria of aged persons, patients with mild cognitive impairment (MCI) and AD, being negatively correlated with the levels of plasmatic antioxidant vitamins and directly proportional to the mini mental state examination score, suggesting a significant contribution of the oxidative damage of aconitase 2 to the energetic imbalance and cognitive dysfunction with increasing age [80]. All of these findings highlight the involvement of oxidative stress in healthy aging and its increase in patients with MCI or AD [81].

Aside from the mitochondrial activity, ROS can be generated by xanthine oxidase, NADPH oxidase, nitric oxide synthase, peroxidases, lipoxygenases, cyclooxygenase, and endoplasmic reticulum [52]. ROS play essential roles in a variety of signal transduction pathways [82]. For example, hydrogen peroxide targets cysteine residues on tyrosine phosphatases, thereby modulating the mitogen-activated protein kinases (MAPKs) pathways. Subsequently, the phosphatidylinositol 3-kinase (PI3K)/Akt pathway [83], the c-Jun N-terminal kinases (JNKs), the p38-MAPK, and the extracellular-regulated kinases (ERKs) pathways are activated [82].

Numerous enzymatic and non-enzymatic cellular systems, called antioxidants, mediate the detoxification functions via the activation of nuclear transcription factors, such as the activator protein 1 (AP-1) transcription factor or the hypoxia-inducible factors (HIFs). However, one of the key regulators mediating the transcription of antioxidant enzymes, including glutathione (GSH), glutathione reductase, glutathione peroxidase (GPx), superoxide dismutase (SOD), catalase (CAT), and heme oxygenase-1 (HO-1) is the nuclear factor erythroid 2-related factor 2 (Nrf2) [84]. Nrf2 belongs to the leucine zipper transcription factors with a short cytoplasmic half-life. It is rapidly sequestered by Keap1 (kelch like erythroid cell-derived protein with CNC homology (ECH)-associated protein 1), which acts as an adaptor protein for a Cullin 3 (Cul3) scaffold protein of Nrf2 ubiquitin ligase (E3) and mediates Nrf2 ubiquitination and degradation by the proteasome [85]. Oxidative modifications at Cys residues of Keap1 change Keap1 conformation and promote its dissociation from Nrf2 [86], which associates with co-activators like CBP (CREB binding protein)/p300 and chromatin remodelers, forms heterodimers with small musculoaponeurotic fibrosarcoma protein, and translocates to the nucleus, binding to ARE (antioxidant response element) sites and initiating the transcription of these antioxidant enzymes [87]. An alternative pathway for the activation of Nrf2 is the phosphorylation of Nrf2 by protein kinase C, casein kinase 2, or MAPKs [88]. Through cross-talk, the Nrf2-ARE pathway indirectly modulates the nuclear factor-kappa B (NF-κB) pathway [82]. NF-κB regulates the transcription of anti-apoptotic proteins and inhibits caspase-dependent cell death pathways, also being a master regulator of inflammation [82]. Moreover, Nrf2 competes with NF-κB for binding CBP/p300, which may contribute to the suppression of the Nrf2/ARE pathway in inflammatory states [55].

Reduced nicotinamide adenine dinucleotide (NADH) is another endogenous antioxidant recently involved in the pathophysiology of neurodegenerative diseases. The main reason for the age-related decline in NAD levels seems to be an increased expression of CD38 [89], a transmembrane glycoprotein that catalyzes the degradation of NAD or its conversion into other metabolites, and also exhibits cyclase activity producing cyclic adenosine diphosphate ribose, a calcium mobilizer that controls neurotransmitter release by neurons and astrocytes [90]. The expression of CD38 was shown to be driven by a series of pro-inflammatory cytokines and chemokines secreted by senescent cells as well as by ROS [91]. The resultant NAD depletion could impair the activity of NAD-dependent enzymes such as poly(ADP-ribose) polymerase (PARP) and contribute to the accumulation of DNA mutations as well as to reduced sirtuin activity [92].

Although oxidative changes occur in all aerobic cells, the brain is particularly prone to oxidative damage due to lower levels of antioxidant enzymes compared to other cell types [93], large membrane surfaces compared to the cytoplasmic volume, and increased content of polyunsaturated fatty acids in cellular membrane [94], as well as to the presence of Cu^+^ and Fe^2+^, transition metals that act as catalyzers in the Fenton reaction leading to hydroxyl generation [95].

ROS act as key second messengers in the innate and adaptive immune response, and ROS overproduction leads to a sustained activation of the inflammatory response [96] and the upregulation of the production of pro-inflammatory cytokines, which, in turn, activate inducible nitric oxide synthase (iNOS) and NADPH oxidase (NOX), promoting the further production of reactive species in a vicious cascade and resulting in the apoptosis of pericytes and the breakdown of the blood–brain barrier [97].

Aside from ROS, the chronic neuroinflammatory state is activated and maintained by a series of pathways: by the PRR pathway, by cytokine signaling pathways, through triggering receptor expressed on myeloid cells 2 (TREM2) signaling [55], and via the cyclic GMP-AMP synthase (cGAS)-stimulator of interferon genes (STING) pathway [98]. The PRRs include membrane receptors, such as toll-like receptors (TLRs), as well as intracellular receptors, such as the nucleotide-binding oligomerization domain (NOD)-like receptors (NLRs), or absent in melanoma 2 (AIM2)-like receptors [99], which bind PAMPs or DAMPs as discussed above, and in the activation of which mitochondria and mitochondria-derived molecules have a crucial role. Neurons are able to detect glial-released pro-inflammatory factors and can either release inhibitory factors leading to the resolution of inflammation or can release DAMPs such as ATP or DNA and further activate glial PRRS [100]. Following th detection of DAMPs, the CARD domain of the NLR (nucleotide-binding oligomerization domain and leucine-rich repeat-containing receptor) binds to the adaptor apoptosis-associated speck-like protein containing CARD (ASC) and procaspase-1, contained in CARD, catalyzes to its active form leading to the production of IL-1β and IL-18 [55], as well as to the cleavage of gasdermin D, which produces pores in the plasmalemma and leads to pyroptosis [101]. NLR family pyrin domain containing 3 (NLRP3) inflammasome activation requires a priming signal inducing NF-κB transcriptional targets [102], after which it relocates together with ASC to the mitochondria and MAMs. TREM2 is a transmembrane protein expressed in microglia that can be activated by the lipids of the cell membrane, lipids in body fluids, or by components of lipoprotein complexes [103]. The gene encoding TREM2 is located at chromosome 6p21.1, together with TREM-like genes (TREML1 and TREML2) [104]. Ligand binding to TREM2 leads to the dephosphorylation of the signaling adaptor protein DAP12, followed by the recruitment and activation of spleen tyrosine kinase (Syk). In turn, Syk activates PI3K, causes Ca^2+^ release from the endoplasmic reticulum, and activates MAP kinases [105]. TREM2 promotes the phagocytosis and clearing of pathogenic proteins and apoptotic cells. Whether TREM2 is important for resident microglia or rather in promoting the infiltration of peripheral myeloid cells is still a matter of debate [106].

The dimeric cGAS protein receptor binds DNA and is activated forming ladder-like networks [107]. Once activated, it produces 2′3′-cGAMP, which binds to STING, an adaptor protein located in the ER. After dimerization, STING translocates to the Golgi apparatus, is phosphorylated by TANK binding kinase 1 (TBK1), and binds to interferon regulatory factor 3 (IRF3), leading to its phosphorylation and activation [108]. Following phosphorylation, IRF3 translocates to the nucleus and activates the transcription of interferons and other cytokines mediating the inflammatory response. Interferon-1 binds to the interferon-1 receptor (IFNAR), with 2 subunits, IFNAR1 and IFNAR2, associated with tyrosine kinase 2 and Janus kinase (JAK1), respectively [109]. After activation, tyrosine kinase 2 and JAK1 activate the signal transducer and activator of transcription 1 and 2 (STAT1 and STAT2), which form a heterotrimeric complex with interferon regulatory factor 9 (IRF9), a complex known as interferon-stimulated gene factor-3 (ISGF-3). This complex, in turn, stimulates the transcription of interferon-stimulated genes, like IL-6, IL-1β, and TNF-α [98]. STING is expressed mainly in microglia but also in astrocytes and neurons [110]. While the mild activation of microglia and astrocytes maintains synaptic plasticity, neurite outgrowth, and neurogenesis [111], the overactivation of glial cells and the excess production of cytokines activate NF-κB and p53, leading to neuronal loss and BBB breakdown [98]. In other words, the accumulation of cytoplasmic DNA during senescence ignites the cGAS-STING pathway, leading to the amplification of neuroinflammation and further cell loss.

Dysfunctional mitochondria are the main source of ROS essential for NLRP3 activation via the non-canonical pathway [112], while mtDNA (and especially oxidized mtDNA) release serves to amplify inflammasome activity [113] and activates the cGAS-STING pathway [114].

To summarize, there is a significant cross-talk between oxidative stress and neuroinflammation: ROS damage biomolecules and activate the redox-sensitive transcription factor NF-κB, initiating the inflammatory response; damage the BBB; and promote the infiltration of peripheral immune cells. Glial inflammatory activation increases the expression of iNOS and NOX, further increasing the production of ROS, and leads to neuronal demise. Dying neurons release DAMPs, additionally activating microglia and triggering NLRP3 inflammasome assembly.

#### 2.1.3. Astrocyte Senescence

Astrocytes are vital for the normal functioning of the CNS, providing nutrients for neurons, regulating synaptic plasticity, modulating the release of neurotransmitters in a Ca^2+^-dependent manner, maintaining ion balance in the extracellular space, and participating in the formation of the BBB [115,116]. Similar to other cell types, astrocytes can initiate a senescence program in response to various stressors [117], leading to the release of chemokines, cytokines, and proteases [118], which further activate astrocytes to an A1-like phenotype [119].

Senescent astrocytes show an increased expression of the *p21^WAF1^* gene, or CIP/KIP (CDK interacting protein/kinase inhibitory protein), which is partly upregulated by p53 and which leads to initial cell cycle arrest by inhibiting CDK2 activity independent of telomere shortening [120]. Further, *p16^INK4A^*, a member of the INK4A family, inhibits CDK4 and CDK6, leading to RB (retinoblastoma protein) hyperphosphorylation and the blockage of the cell cycle entry to the S phase [121]. In addition, senescent astrocytes exhibit nuclear enlargement and changes in the nuclear morphology, as well as alterations in the integrity of the nuclear envelope caused by the downregulation of lamin B1 and other nuclear lamin proteins [122]. Ultrastructurally, there are chromatin alterations and the formation of senescence-associated heterochromatic foci, which associate with the diminished expression of proliferation-promoting genes and lead to irreversible cell cycle arrest [123].

In addition to morphologic changes (hypertrophic somata and processes), astrocytic activation leads to the upregulation of glial fibrillary acidic protein (GFAP), as well as to calcium dyshomeostasis and the upregulation of Ca^2+^-signaling mediators such as L-type voltage-sensitive calcium channels, ER Ca^2+^-release channels, or Ca^2+^-binding proteins [124]. Aside from modulating the activity of several transcription factors, such as NF-κB, peroxisome proliferator-activated receptors (PPARs), or the JAK/STAT pathway, Ca^2+^ also activates calcineurin, a phosphatase that dephosphorylates and activates the receptors of nuclear factor of activated T cells (NFATs) [124]. Glial calcineurin/NFAT activity leads to the upregulation of key mediators of inflammation, such as TNF-α, IL-6, or cyclooxygenase 2 [125], and modulates the expression of excitatory amino acid transporters (EAATs), leading to the decreased uptake of glutamate by astrocytes, as will be discussed further [126].

The number of mitochondria increases, likely due to impaired mitophagy in astrocytes as well [127], but their membrane potential is altered, leading to increased ROS production and the release of mtDNA. Other organelles, such as lysosomes, tend to accumulate, and the lysosomal enzymes, such as senescence-associated beta galactosidase (SA-β-Gal), are upregulated, making SA-β-Gal a common marker of senescent cells [117].

Increased oxidative stress in astrocytes causes transcriptomic changes, upregulating genes associated with proinflammatory cytokines via the p38/MAPK and NF-κB pathways [119,128], as well as by HMGB1 (high-mobility group B), another regulator with increased expression in aging astrocytes. HMGB1 is able to increase the efficiency of NF-κB transactivation by interacting with NF-κB complexes, thereby strengthening the pro-inflammatory response and increasing the production of IL-6, IL-8, chemokines, and proteinases, collectively known as the senescence-associated secretory phenotype (SASP) [129].

As stated, astrocytes regulate neuronal function through the uptake of released excitatory and inhibitory neuromediators, such as glutamate or γ-aminobutyric acid (GABA). They express excitatory amino acid transporters (EAATs) 1 and 2 [130] for the uptake of excess glutamate, which is converted by glutamine synthase into glutamine [131]. Both glutamine synthase levels and the expression of EAAT1 decrease with age in astrocytes [132]. Moreover, the activity of glutamine synthase is very sensitive to oxidative stress, decreasing the available metabolic substrates to neurons and contributing to excitotoxic neuronal death [119].

Another role of astrocytes in the CNS is to synthesize cholesterol, an essential component of cell membranes, due to the expression of apoE and SREBP2 (sterol regulatory element-binding protein 2), a transcription factor that regulates the expression of 3-hydroxy-3-methylglutaryl CoA reductase (HMGCR). Given the presence of the BBB, the brain cholesterol content is largely independent of dietary intake [133]. In senescent astrocytes, the expression of cholesterol synthesis-associated genes and HMGCR is decreased, leading to a dysregulation of cholesterol synthesis and decreased synaptic support of neurons [119].

#### 2.1.4. Neuroinflammation and Defective Autophagy

Under normal conditions, cells prevent the accumulation of protein aggregates and damaged organelles by autophagy. Specifically for mitochondria, the process is called mitophagy [39,134].

The formation of the autophagosome starts with the activation of the Unc-51-like kinase (ULK) complex, which contains ULK1, ULK2, ATG13 (autophagy-related protein 13), ATG101, and focal adhesion kinase (FAK) family-interacting protein of 200 kDa (FIP200). The activation of the ULK complex occurs through phosphorylation by 5′-adenosine monophosphate (AMP)-activated protein kinase (AMPK), while mammalian target of rapamycin (mTOR) complex 1 inhibits ULK [135]. Following activation, ULK complex recruits class III phosphatidylinositol 3-kinase (PI3K) complex I (containing vascular protein sorting (VPS) 34, VPS 15, beclin1, and ATG14), which will lead to the generation of phosphatidylinositol 3-phosphate (PI3P) on the phagophore [135] and the recruitment of PI3K-binding proteins and tryptophan-aspartic acid (WD) repeat domain phosphoinositide-interacting (WIPI 1 and 2). These events are followed by the recruitment of the ATG12-ATG5-ATG16L1 complex. Subsequently, ATG4 cleaves the light chain 3 (LC3) family member proteins, the cleaved fragment (LC3-I) being conjugated to the phagophore membrane via ATG7, ATG3, and the ATG12-ATG5-ATG16L1 complex [136], contributing to phagophore membrane elongation and closure. Completed autophagosomes migrate along microtubules to the lysosomes located in the perinuclear region. The tethering of the autophagosome to the lysosomal membrane is mediated by a series of tethering factors, after which soluble N-ethylmaleimide sensitive factor attachment protein receptors (SNAREs) mediate the fusion of the two membranes [137]. About 40 SNARE proteins have been identified in mammalian cells, with key functions in intracellular membrane fusion. Depending on on the identity of the amino acid located at the center of their 60-amino-acid-long eponymous domain, these proteins can be classified into Q-SNAREs and R-SNAREs [138]. For vesicle fusion, an R-SNARE (also known as v-SNARE) located on the membrane of one vesicle forms a *trans*-SNARE complex with three different Q-SNAREs (also called t-SNAREs) located on the target vesicle’s membrane (containing Qa, Qb, and Qc SNARE motifs), leading to the fusion of the two membranes. Consequently, all SNAREs come to be located on the same membrane, and the *trans*-SNARE complex transforms into a *cis*-SNARE complex, which is recognized and disassembled by N-ethylmaleimide sensitive fusion protein (NSF) and alpha soluble NSF-attachment protein [139]. The v-SNARE undergoes retrograde transport and is recycled to the donor compartment, while the t-SNARE subunits are recognized for future fusion events [140].

Although low levels of ROS induce autophagy, increased oxidative damage of proteins, and the age-related reduced expression of LC3 and PINK1 (necessary for mitophagy), mainly in women [141], cause an impairment of autophagy and leads to the accumulation of damaged organelles and altered proteins. In addition, the accumulation of DNA and mtDNA damage impairs the transcription of the discussed proteins involved in autophagy and leads to the accumulation of p62, which further impairs the DNA damage responses [142] and induces NF-κB activity, activating inflammation [143]. Basically, all the damaged cellular components accumulated as a result of impaired autophagy are released and recognized as DAMPs and activate TLR9, triggering the production of IL-6, IL-8, IL-15, IL-1β, TNF-α, ICAM1 (intercellular adhesion molecule 1), matrix metalloproteinases, and MCP-1 (monocyte chemotactic protein-1), collectively known as SASPs [25] and which are able to reinforce cellular senescence [144].

## 3. Neuroinflammation in Alzheimer’s Disease

Already in the original description of the disease in 1907, Alois Alzheimer noted the presence of the neurofibrillary tangles, later shown to consist of cleaved and hyperphosphorylated intracellular protein tau aggregates and of neuritic plaques (originally called “miliary foci”), consisting of a “special substance in the cortex” surrounded by dystrophic neuronal processes [145]. The “special substance” was characterized in 1984 by Glenner and Wong to be a peptide with 40 or 42 amino acids [146], originating from the amyloid precursor protein (APP) identified in 1987 [147].

**Figure 1:**
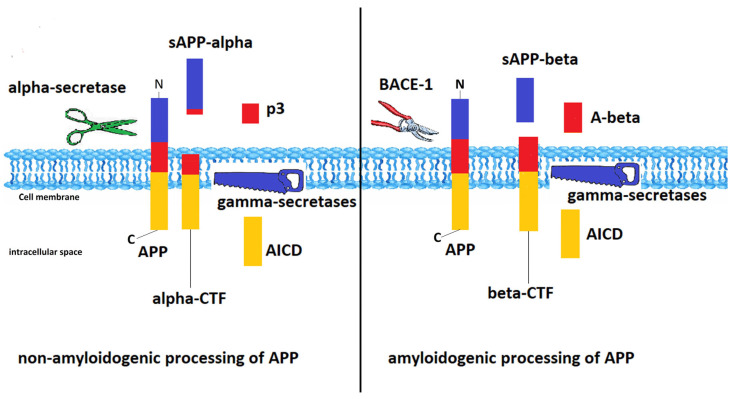
The processing of APP. APP has a large N-terminal ectodomain and a shorter C-terminus domain. The Aβ peptide starts in the ectodomain and continues in the transmembrane region (pictured in red). Alpha-secretase cleaves APP within the Aβ domain and thus does not lead to generation of Aβ. The soluble sAPPα fragment is released into the extracellular space. Both α- and β-secretase (BACE-1) cleavage of APP is followed by γ-secretase processing of the C-terminal fragment (CTF) residue, resulting in an identical intracellular C-terminal fragment (AICD). BACE-1 cleavage leads to the release of sAPPβ, while further processing of the β-CTF will lead to generation of Aβ.

APP is a single-pass transmembrane protein secreted in large amounts by neurons and rapidly metabolized, the precise function of which is yet elusive. Alternate splicing of the APP transcript generates eight isoforms, of which the 695 amino acid form is expressed mainly in the brain [145]. After sorting in the ER and Golgi apparatus, APP is delivered to synaptic terminals [55] and presented at the cell surface, where it can be further processed or reinternalized via a clathrin-mediated process. APP is cleaved either by α- or β-secretases. Alpha-secretases are members of the ADAM (a disintegrin and metalloproteinase) family of proteases and cleave APP within the Aβ sequence, generating a soluble APP fragment (sAPPα) that remains in the extracellular compartment and modulates neuronal excitability and synaptic plasticity and an 83-amino-acid carboxy-terminal fragment, further processed by the γ-secretase complex to an intracellular C-terminal fragment and an extracellular p3 fragment [145]. The cleavage of APP by β-site APP cleaving enzyme (BACE-1) gives rise to a soluble sAPPβ ectodomain and a 99-amino-acid C-terminal membrane-bound fragment, which, after cleavage by γ-secretase, generates Aβ fragments with 38 to 44 amino acids and an intracellular C-terminal fragment [145,148]. The different Aβ fragments have various degrees of toxicity. Aβ40 and Aβ42 play a key role in the aggregation of neuritic plaques, with Aβ42 being the most prone to aggregation. Familial forms of AD have an increased Aβ42:Aβ40 ratio [148,149]. Being the only β-secretase responsible for Aβ production, BACE1 is the rate-limiting enzyme for amyloid-β peptides generation and plays a key role in AD pathogenesis. Both protein and BACE1 mRNA levels are abnormally elevated in post mortem brain tissues from AD patients [150,151]. The expression of BACE1 is regulated by complex mechanisms at both the transcription and translational levels. For example, activators of the Nrf2/ARE pathway not only induce the expression of antioxidant genes but also reduce BACE1 expression [152]. γ-secretase is a protein complex composed of presenilin (PSEN) 1 or 2, two multipass transmembrane proteins (Aph-1 and Pen-2), and a transmembrane glycoprotein (nicastrin) [145]. The amyloidogenic and non-amyloidogenic processing of APP is shown in Figure 1.

The reinternalization of APP into endosomal compartments containing BACE-1 and γ-secretase will result in intracellular Aβ generation, which is dumped into the extracellular space or degraded in lysosomes. SorLA is an adaptor protein belonging to the low-density lipoprotein receptor superfamily that binds intracellular APP and shuttles it from endosomes to the Golgi apparatus, preventing the excessive intracellular generation of Aβ [153]. Aβ peptides aggregate into a beta-sheet conformation and form oligomers, protofibrils, and fibrils [154]. Due to the increased hydrophobicity of its C-terminus domain, Aβ42 is more prone to aggregation into 7 nm diameter fibrils of two twisted protofilaments of Aβ42 monomers [155]. Aβ fibrillization can “seed” in a prion-like fashion, but some evidence suggests that aggregation and seeding may require Aβ uptake by microglia [156].

The main function of tau protein is to stabilize the microtubule tracks along which the axonal transport of vesicles and organelles occur. The hyperphosphorylation of the protein, as seen in AD, leads to the detachment of tau from the microtubules, which become disorganized and unstable, and intracellular aggregation in the form of neurofibrillary tangles. For a while, it has been considered that the two pathways act independently to promote AD pathology. However, recent research has shown that Aβ, either as plaques or soluble oligomers, initiates a pathological cascade that leads to tau misfolding and aggregation [157].

Cortical plaques are widespread 10 to 20 years before the emergence of the clinical picture of AD and can be found in up to 40% of cognitively normal elderly persons [158], while tau aggregates are commonly found in the medial temporal lobe of persons older than 60 years [159]. Although Aβ can directly promote tau oligomer formation and enhance tau aggregate seeding [160], they rarely co-localize at synapses [161], which argues against a major role for the direct physical interaction of the two pathological proteins. It appears that the link leading to the synergistic action of Aβ and misfolded tau is microglial activation, elicited by both Aβ and tau overexpression [157]. Microglia is able to take up seed-competent tau and decompose it at the expense of undergoing activation [162] and increased release of pro-inflammatory cytokines. Bystanding microglia may also become reactive and form somatic junctions with neurons, thereby contributing to tau seeding between cells [163].

Genetic studies also support the role of microglia and neuroinflammation as a major contributor to AD pathogenesis. The presence of the *APOE* ε4 allele increases the risk of AD 3- to 4-fold [164]. Rare variants in the *SPI1, BIN1, INPP5D, ABCA7, SORL1, MS4A, CD2AP,* or *PICALM* genes, as well as mutations in *PSEN1* and *PSEN2,* have been shown to influence the risk of developing AD [165,166]. Protective genes, such as the *APOE* ε2 allele, *APOE* ε3 allele (Christchurch mutation), the Ala673Thr Icelandic protective mutation of *APP,* or a rare Pro522Arg amino acid change in the *PLCG2* gene have been shown to decrease the risk of AD [164]. Overall, at present, a polygenic risk score can be calculated that enables us to identify patients at risk for Alzheimer’s disease with 75–85% accuracy [167]. The further identification of Alzheimer’s-disease-associated genetic variants of *TREM2* Arg47His, Arg62His, and Asp87Asn, which decrease the binding of TREM2 to ApoE, as well as the identification of other proteins (SHIP1, CD2AP, RIN3, BIN1, PLCG2, CASS4, and PTKB2) associating with an increased risk of AD via modulating endocytosis, motility, and phagocytosis in microglia, suggest a central role of the latter in AD pathogenesis. In addition, vascular and endothelial dysfunction, weakening of the BBB [168], dysfunction of the meningeal lymphatic system [169], peripheral inflammation [170], and alterations of the gut microbiota [171] may all contribute to the clinical development of AD.

### 3.1. The Role of Microglia in Alzheimer’s Disease Pathogenesis

One of the main functions of microglia is to detect and clear toxic protein aggregates. Unfortunately, with age and with the progression of AD, microglia become dysfunctional and dystrophic, known as “dark microglia”, and fail in this task. Amyloid compaction exposes microglia to Aβ and contributes to microglial activation via TLR and NOD-like receptor signaling [172,173], leading to proinflammatory cytokine production. The precise mechanism by which tau activates microglia is still unclear, but a recent study showed that, following microglial uptake and lysosomal sorting, aggregated tau can activate the NLRP3-ASC inflammasome [174]. In addition, polyglutamine binding protein 1 (PQBP1) interacts with tau 3R/4R proteins and is able to trigger the innate immune response via the activation of the cGAS-STING pathway [175]. Several receptors have been involved in microglia dysfunction.

#### 3.1.1. TREM2 Signaling

In the human genome, the gene encoding TREM2 is located within a cluster of genes at chromosome 6p21.1 [104]. TREM2 is a transmembrane protein expressed in microglia and other immune cells with an ectodomain, a transmembrane domain, and a cytoplasmic tail [176]. It is activated by lipids (from cell membrane or from body fluids) or by lipoprotein complexes [103] including lipidated and non-lipidated ApoE.

Proteolytic processing cleaves the ectodomain of TREM2 and releases it as a soluble fragment (sTREM2) with pro-inflammatory actions [177] that can be detected in the CSF and serum of patients (elevated in AD) [178]. Upon ligand binding, its cytoplasmic tail recruits the DNAX-activation protein of 12 kDa (DAP12), followed by the activation of Syk, further downstream effects resulting in activation of MAP kinases, GSK3β [179], and PI3K, which leads to the Ca^2+^ release from the ER [105].

The expression of TREM2 increases with age and has been found to increase even more in patients with AD [179], while Aβ binding to the TREM2 ectodomain enhances the interaction of the receptor with its ligands, promoting the survival of microglia via the activation of the canonical Wnt signaling pathway [180]. This is likely a compensatory mechanism in response to the presence of Aβ, as TREM2 deficiency leads to reduced ATP levels and mTOR activity and increased ULK1 and AMPK activity and also promotes microglial autophagy [181]. Moreover, mice with reduced TREM2 expression showed longer amyloid filaments (as revealed by the ultrastructural analysis of amyloid plaques with stochastic optical reconstruction microscopy), suggesting that microglia reduce the exposure of neuronal processes to neurotoxic species of Aβ and limit neuritic dystrophy by compacting amyloid fibrils [182].

#### 3.1.2. Scavenger Receptor Class A (SR-A) in Alzheimer’s Disease

Scavenger receptor class A (SR-A) is a phagocytic pattern recognition receptor expressed primarily on microglia and astrocytes and is involved in pathogen and apoptotic cell clearance, lipid transport, intracellular cargo transport, and cellular adhesion [183]. Although in mouse models of AD, its expression is increased in early stages, paralleling the increased microglial uptake of Aβ, in later stages of AD, as well as with aging, the expression of SR-A is reduced, leading to increased Aβ deposition [184]. While AD mice lacking SR-A receptors showed decreased microglia-mediated phagocytosis of soluble Aβ, the overexpression of the receptor increased Aβ clearance [185], making SR-A a possible target in the treatment of AD.

#### 3.1.3. CD33 Receptor in Alzheimer’s Disease

CD33 is a type I transmembrane glycoprotein [186] expressed on the surface of microglial cells and peripheral monocytes. CD33, and especially the single-nucleotide polymorphism rs3865444C, have been identified by genome-wide association studies (GWAS) as one of the prominent risk factors for AD [186]. The intracellular C-terminal of CD33 contains two immune-receptor tyrosine-based inhibition motifs (ITIMs) that inhibit signal transduction in cells and can also inhibit DAP12 and TREM2 signaling [187]. In addition, its extracellular N-terminal domain can bind sialylated glycoproteins and glycolipids on amyloid plaques, preventing the efficient clearance of Aβ by microglia [183].

#### 3.1.4. CD36 Receptor in Alzheimer’s Disease

CD36 is a member of the class B scavenger receptor family that promotes microglial migration toward Aβ deposits and Aβ phagocytosis, but the interaction of the receptor with Aβ activates microglia and leads to the release of pro-inflammatory mediators via the activation of Src phosphotyrosine kinases Lyn, Fyn, and p44/42 mitogen-activated protein kinase [188], as well as to the activation of the NF-κB pathway [189].

#### 3.1.5. Complement Receptor 3 (CR3) in Alzheimer’s Disease

CR3 is a dimeric receptor comprising CD18 and CD11b [190] expressed mainly in microglial cells, which binds and targets damaged cells and cell debris to microglial clearance but which is also involved in synaptic pruning during development [191]. Oligomeric Aβ upregulates the expression of C1q, C3, and CR3 and contributes to the activation of the complement cascade, driving through the CR3 microglia-dependent elimination of synapses [192]. CR3s appear to play a dual role in AD pathogenesis: although they promote the phagocytosis of fibrillary Aβ, they also potentiate synaptic dysfunction and neuronal loss, while, via reducing the expression and activity of Aβ-degrading enzymes, such as matrix metalloproteinases (MMP2, MMP9), they may indirectly inhibit the degradation of soluble Aβ [183].

#### 3.1.6. TNF Signaling in Alzheimer’s Disease

TNF-α binds to 2 receptors: a 55 kDa TNF receptor 1 (TNFR1), expressed in all cell types, and a 75 kDa TNF receptor 2 (TNFR2), expressed mainly in cells of the immune system and endothelial cells. TNFR1 contains an intracellular death domain and binding of TNF to TNFR1 promotes the recruitment of the FAS-associated death domain and the subsequent activation of caspases 8 and 3, leading to apoptosis. The binding of TNF to TNFR2 promotes neuroprotective and regenerative pathways via interaction with a series of TNF receptor-associated factors and cellular inhibitor of apoptosis proteins, leading to the activation of MAP kinases and the Akt and the NF-κB pathways [55].

All the aforementioned receptors exhibit age-related changes in expression: SR-A and CD36 are decreased, whereas TREM2, CD33, and CR3 are upregulated. The chronic stimulation of microglia promotes microglial dystrophy and decreases the ability of microglial cells to protect adjacent neurons [193]. The pro-inflammatory cytokines released by activated microglia reduce the expression of Aβ-binding receptors and Aβ-degrading enzymes, leading to increased Aβ accumulation. As for tau protein, although microglia are able to phagocytose tau, they cannot degrade it and may even contribute to tau seeding, as discussed above [194]. These dysfunctions occur on top of the normal age-induced alterations of microglial morphology, with lower motility and fewer ramifications [195], and the age-associated SASP.

**Figure 2:**
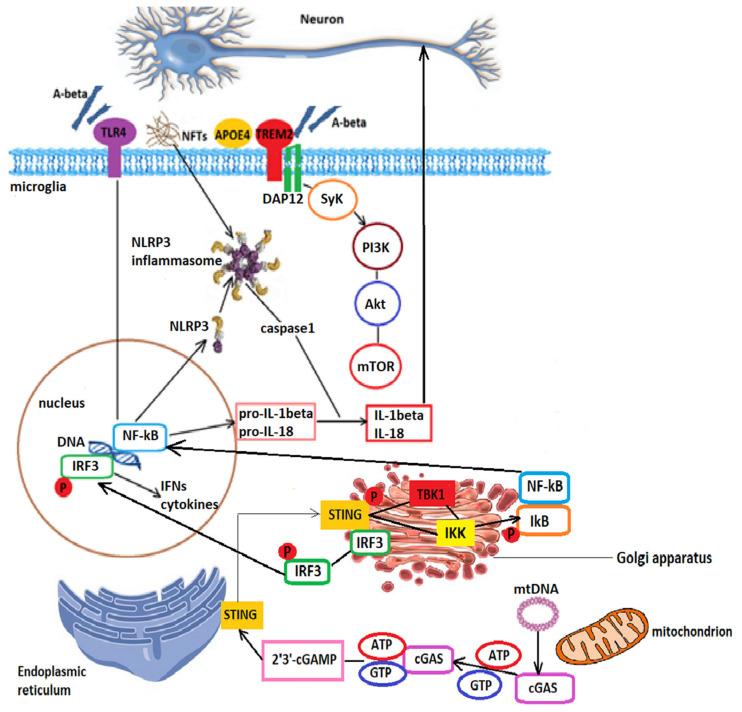
Aβ binds to Toll-like receptors (TLR) and TREM2, while both Aβ and tau fibrils (NFTs) can trigger the NLRP3 inflammasome assembly. Ligand binding to TREM2 activates DAP12 through charge interactions in their transmembrane domain, followed by recruitment of spleen tyrosine kinase (Syk) and activation of phospoinositide 3-kinase (PI3K), which targets Akt and activates mammalian target of rapamycin (mTOR), inhibiting autophagy and impairing Aβ clearance. Mitochondrial DNA (mtDNA) activates cGAS, which synthesizes cGAMP that binds to STING. Subsequently, STING translocates to the Golgi apparatus and is phosphorylated by TANK binding kinase 1 (TBK1), followed by binding to interferon regulatory factor 3 (IRF3), which is also phosphorylated and activated by TBK1. Phosphorylated IRF3 translocates to the nucleus, where it promotes the production of interferons (IFNs) and cytokines that enhance the inflammatory response. TLR signaling and phosphorylated STING can also activate IκB kinase (IKK), resulting in phosphorylation of the inhibitor of κB (IκB) and release of NF-κB, the master transcription factor regulating the production of pro-inflammatory cytokine precursors and the NLRP3 inflammasome assembly. Caspase-1, contained in the NLRP3 inflammasome, cleaves the precursors of pro-inflammatory cytokines, resulting in IL-1β and IL-18, which can damage neurons.

Figure 2 shows the complex interactions leading to microglial activation and its consequences in AD.

The myriad of cytokines, chemokines, and growth factors that undergo changes in the CNS as a consequence of the accumulation of Aβ and tau hyperphosphorylation can act either to enhance AD pathology or exhibit a protective effect, as shown in Table 1.

In conclusion, if in pre-clinical stages of AD, microglia phagocytose both Aβ and tau, following Aβ accumulation, microglia become dysfunctional and releases tau seeds and pro-inflammatory cytokines, creating an inflammatory environment that has a major contribution to neuronal and synaptic loss [183].

**Figure 3:**
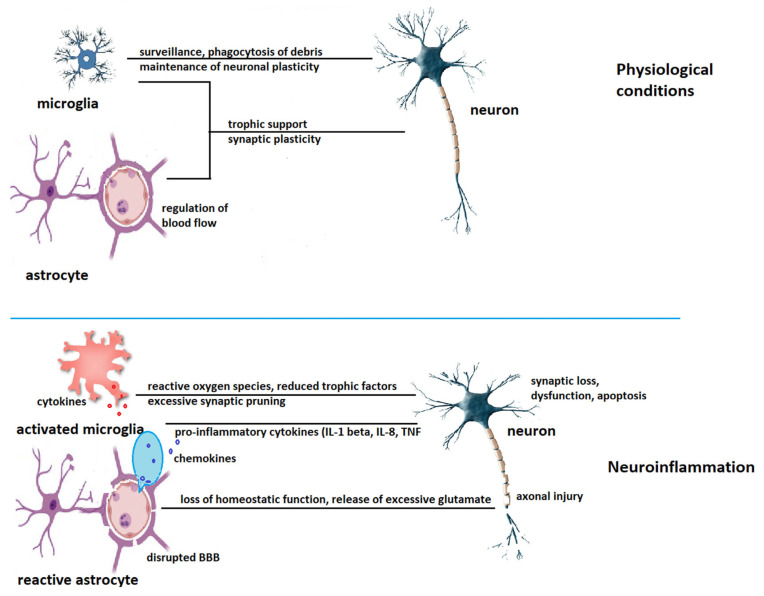
The various cytokines and chemokines released during neuroinflammatory states lead to synaptic loss, excitotoxicity, oxidative damage, and culminate with neuronal loss.

Figure 3 shows the homeostatic function of microglia and astrocytes under physiological conditions and the disrupted cooperation with neurons during neuroinflammation.

### 3.2. Mitochondrial Dysfunction and Neuroinflammation in Alzheimer’s Disease Pathogenesis

Compelling evidence implicates neuronal mitochondrial dysfunction in the pathogenesis of neurodegenerative diseases [39], including AD, up to the point of formulating the “mitochondrial cascade hypothesis” in 2004 [219]. Increased pro-inflammatory cytokine levels such as TNF and IL-1β, as occurs in neuroinflammatory states, as well as ROS have been shown to reduce the activity of pyruvate dehydrogenase and alpha-ketoglutarate dehydrogenase in the tricarboxylic acid cycle and promote post-translational modifications that further decrease the activity of these enzymes [220]. Regarding mitochondrial OXPHOS, exposure to TNF reduces complex I (cytochrome c oxidase) and complex V (ATP synthase) activity and decreases ATP levels, one of the mechanisms being an increase in microRNAs targeting transcripts coding for complex I and V subunits [221]. In addition, the transcription of ETC genes is impaired through reduced PGC-1α mRNA caused by TNF-α [222]. The increased activities of hexokinase (the glycolysis-initiating enzyme) and of lactate dehydrogenase in subcortical regions of patients with AD suggest a switch to anaerobic metabolism to compensate for the inefficient aerobic one [223]. In turn, the reduced ETC activity results in the increased production of ROS, which irreversibly damages ETC complexes and attacks mtDNA, further compromising OXPHOS. In the advanced stages of AD, brain samples from patients show significant nitration and lipoxidation of ATP synthase, as well as oxidative alterations of aldolase, glyceraldehyde-3-phosphate dehydrogenase (GAPDH), α-enolase, and phosphoglycerate mutase 1 (PGAM1) [224,225,226]. Cellular apoptosis leads to the extracellular release of molecules that are recognized as DAMPs by microglia, activating the latter and leading to the production of significant amounts of ROS via the NOX-mediated oxidative burst [227].

The two pathological proteins of AD, Aβ and hyperphosphorylated tau, contribute to mitochondrial dysfunction as well. APP (both full length and C-terminal fragments) and Aβ are associated with the mitochondrial membrane in human brain regions affected by AD. This is thought to block mitochondrial import channels and interfere with the assembly of the ETC [228]. ROS and reactive nitrogen species peroxidize membrane lipids and oxidize intracellular proteins and nucleic acids [229], promoting APP proteolysis and Aβ generation [230] in a vicious cycle. Specific mitochondrial-binding partners for Aβ, such as Aβ-binding alcohol dehydrogenase, exacerbate Aβ toxicity and free radical generation [231]. Hyperphosphorylated tau dislodges from microtubules and increases its affinity for other tau monomers, forming oligomers that potentiate neuronal damage and synaptic loss [232]. As the oligomers lengthen, they adapt a β-sheet structure and transform into granular aggregates, which then fuse to form tau fibrils and neurofibrillary tangles [233]. Of these various tau species, oligomers appear to be the most toxic ones, altering the mitochondrial membrane, diminishing complex I activity, and activating caspase-9 [234,235]. In vivo imaging studies using fluorodeoxyglucose PET have shown regional low glucose consumption in the cerebral areas of AD patients affected by the characteristic pathology [236].

PGC-1α, the master regulator of mitochondrial biogenesis, is abundantly expressed in tissues with high energy demand but has been shown to decrease in both AD patients and transgenic mouse AD models [237]. Most of the mitochondrial proteins are encoded by nuclear DNA and must be imported mainly through the translocase of the outer membrane (TOM), which consists of a pore-forming protein TOM44 and three receptor proteins on the cytosolic side (TOM20, TOM22, and TOM70) [238]. The reduced expression of TOM22 and TOM70 [239] and the association of Aβ with the mitochondrial membrane augments the inhibition of the protein import system [240]. APP can also form stable complexes with translocases of the OMM and IMM, while Aβ is able to translocate to mitochondria and localize to the cristae, further impairing the import of essential mitochondrial proteins [238].

A physiological pool of healthy mitochondria depends on a proper balance between mitochondrial fusion and fission. Biopsy samples from AD patients revealed altered morphology with the excessive fragmentation of mitochondria in pyramidal neurons [241], as well as a peculiar shape, termed “mitochondria-on-a-string”, consisting of teardrop-shaped mitochondria (0.5 μm in diameter) connected by a thin double membrane [242,243], suggesting fission arrest. Biochemical analyses found reduced expression of OPA1 and mitofusins and increased levels of Drp1 and Fis1 in AD brains [244]. Because both Drp1 and Mfn2 are substrates for calpain, the reduced levels of these GTPases could be due to calpain cleavage [245]. Moreover, the Aβ-induced S-nitrosylation of Drp1 can increase its translocation to mitochondria [246]. In later stages of AD, Aβ-Drp1 interactions are increased by Drp1-hyperphosphorylated tau complexes, increasing mitochondrial fission [247].

Calcium signaling and oxidative stress significantly contribute to Aβ-induced mitochondrial fragmentation. Aβ increases mitochondrial calcium influx and promotes calcium-/calmodulin-dependent protein kinase II (CAMKII)-mediated protein kinase B (Akt) activation, leading to Drp1 phosphorylation and mitochondrial translocation [248], as does the ROS-mediated activation of extracellular signal-regulated kinase (ERK) [249]. The opposite process, mitochondrial fusion, requires the maintenance of the mitochondrial membrane potential for post-translational OPA1 processing [250]. Normally, tau stabilizes the actin cytoskeleton and disrupts the physical association of Drp1 and mitochondria, thereby preventing excessive fission. Hyperphosphorylated tau leads to the disturbance of the microtubule network and indirectly promotes mitochondrial fission. Experimentally, the genetic ablation of tau in mice resulted in decreased fission, a reduced rate of ROS production, and the enhanced generation of ATP [251].

Irreversibly damaged mitochondria are disposed through mitophagy, a process that begins with the recruitment of PINK1 and Parkin on the OMM. Mitophagy is altered in AD; the research shows swollen mitochondria with distorted cristae in the biopsy samples of human AD patients and transgenic animal models of AD [252]. The accumulation of tau via the interaction of its projection domain with Parkin sequesters the latter in the cytosol [253]. In addition, by altering the mitochondrial membrane potential, tau impairs Parkin and PINK1 recruitment to the OMM [254]. Moreover, lysosomes are located mainly in the cell body and proximal dendrites, whereas mitochondria are distributed along axons and dendrites and must be trafficked to the cell soma in order to undergo mitophagy [255]. Although axonal autophagosomes form around damaged mitochondria, research has shown that they do not simply fuse with nearby lysosomes but rather undergo a maturation process while being transported to the cell body. The fusion of these autophagosomes with endosomes initiates the dynein-mediated microtubule-dependent retrograde transport [256], a process during which these autophagosome–endosome hybrids (amphisomes) acquire lysosomal proteins such as LAMP1 and multiple lysosomal proteases in parallel with their acidification [257]. This process is clearly disturbed in AD, wherein amyloid plaques are surrounded by neuritic swellings identified as axons filled with lysosomes with a low content of hydrolases [258]. The disturbed lysosomal distribution may be related to dysfunctions in JIP3 or snapin [255].

Mitochondrial trafficking is two-directional. Healthy mitochondria, with high membrane potential, are moved toward synaptic sites via anterograde transport, while mitochondria with impaired membrane potential are trafficked in a retrograde direction toward the cell soma [55]. Anterograde transport is mediated via kinesin-1, its heavy chain interacting with the atypical Rho GTPase Miro and with Milton to bind its C-terminus. The main protein responsible for retrograde transport is dynein, which interacts with dynactin, as well as with Miro and Milton, to perform this task [259]. The dephosphorylation of Miro and Milton by PINK1 and Parkin leads to the detachment of kinesin and mitochondrial arrest [260]. Aβ reduces the expression of kinesin [261] and impairs the function of dynein by interacting with its intermediate chain [262]. The overexpression and hyperphosphorylation of tau enhance mitochondrial binding to microtubules [263], lead to the disassembly of the microtubule tracks, and sequester the c-Jun N-terminal kinase-interacting protein 1 (JIP1), which associates with the kinesin motor protein complex [253] in the cell body [264].

As for the Ca^2+^-buffering function of mitochondria, both presenilin 1 (PSEN1) and presenilin 2 (PSEN2) localize at MAMs [265] and interact with RyRs to increase Ca^2+^ release from the ER [266]. Aβ aggregates are able to form calcium-permeable channels in membranes [267] and can mediate Ca^2+^ transfer from ER to the mitochondria through the MCU [268], while tau inhibits mitochondrial calcium efflux [269]. In turn, excess cytosolic Ca^2+^ augments tau hyperphosphorylation leading to tau detachment from microtubules, tau misfolding and aggregation, and neurofibrillary tangle formation [53].

Chronic inflammation also alters mitochondrial dynamics leading to an irregular distribution and impaired morphology of mitochondria in neurons. Exposure to TNF-α increases the expression of both Fis1 and OPA1 [270].

Recent research has highlighted the contribution of microglial mitochondrial dysfunction to Alzheimer’s disease pathogenesis. Microglia respond to and engulf Aβ via receptors expressed on the cell surface (CR3, RAGEs, TLRs TREM2), leading to microglial activation and the release of pro-inflammatory cytokines [172]. Internalized Aβ interacts with mitochondrial calcium uniporter (MCU), leading to mitochondrial Ca^2+^ overload and the reduction of the mitochondrial membrane potential [271] as well as ER stress [as shown by elevated levels of CHOP (C/-EBP homologous protein)]. This enhances ROS production. Moreover, the interaction of Aβ with P2X_7_ receptors leads to the activation of NF-κB pathway and of NLRP3, causing the release of cytochrome c and microglial apoptosis [272]. Impaired TREM2 signaling leads to the deficient activation of the mTOR pathway and enhanced microglial autophagy, resulting in a decreased number of mitochondria and potentiating energy deficiency [181].

### 3.3. Impaired Autophagy in Alzheimer’s Disease

Autophagy plays a crucial role in abnormal protein clearance, along with the ubiquitin–proteasome system. The accumulation of abnormal subcellular vesicles in swollen or dystrophic neurites identified as immature autophagic vacuoles [273] indicates an impairment of this process. Although the exact mechanisms are still under research, it appears that the expression of beclin-1, necessary for the initiation of autophagy, is reduced due to the increased activity of caspase-3 [274]. A decreased expression of p62, an autophagic cargo receptor, was also reported [275]. However, more recent research has shown that autophagy is actually upregulated in AD due to the transcriptional upregulation of positive regulators of autophagy as well as to the reactive-oxygen-species-dependent activation of type III PI3 kinase, a critical kinase for the initiation of autophagy, and of ATGs [276]. The retrograde transport of these vesicles, a process in which tau plays a critical role, is also severely impaired [277]. In addition, tau hyperphosphorylation may lead to lysosomal aberrations [278]. The finding that tau deficiency protects against Aβ toxicity suggested that tau may be subject to a toxic gain of function in AD [279], although other researchers argue that axonal dysfunction may be caused by abnormal lysosomal proteases [280]. Further studies are needed to clarify the molecular defects underlying the failure of the transportation of autophagic vesicles in AD [281,282].

In addition, Aβ hinders the fusion of autophagosomes with lysosomes. Sharma et al. demonstrated for the first time the reduced formation of SNARE complexes in AD post mortem tissue samples [283]. Due to the unaltered expression of individual SNARE proteins, they ascribed this finding to Aβ hindering the “zippering” of v-SNARE VAMP-2 with t-SNAREs syntaxin-1 and SNAP-25. Subsequent research in APP-PS1 transgenic mice demonstrated that Aβ42 oligomers interact with the t-SNARE syntaxin 1a with high affinity, disrupting its association with VAMP-2 [284]. To date, a series of SNAREs have been shown to be involved in AD pathogenesis, such as the v-SNAREs syntaxin-1-5, -7, -11, -16-18, GS-15, GS-27, GS-28, Vti1a and Vti1b, BET1, SNAP-23, SNAP-25 and SNAP-29, or the t-SNAREs VAMP-1, -2, -3, or VAMP-8 [285].

### 3.4. Senescent Astrocytes and Alzheimer’s Disease

Several studies have shown that altered astrocytic dysfunction is involved in the onset and progression of AD [286,287]. The cells have important contributions to Aβ clearance and degradation via the low-density lipoprotein receptor-related protein 1 (LRP1) and scavenger receptor B1 (SR-B1) [288], receptors whose expression is reduced in aged astrocytes [289]. In addition, amyloid plaques are surrounded by SA-β-Gal-positive astrocytes, while Aβ_42_ is able to induce astrocytic APP and BACE1 processing, thereby further increasing oligomeric and fibrillary Aβ [290]. Astrocytes also play a crucial role in tau hyperphosphorylation and NFT formation, as shown by Bussian et al. [291], who demonstrated that the removal of senescent astrocytes and microglia significantly reduced the deposition of hyperphosphorylated tau in a transgenic mouse model.

As for the role of astrocytes in modulating synaptic function and neural transmission, hippocampal neurons co-cultured with senescent astrocytes exhibited a reduced size of synaptic vesicles and an impaired synaptic maturation and transmission [292]. Furthermore, the release of SASP factors, such as IL-6 and the diminished production of neurotrophins (BDNF, NGF, insulin-like growth factor 1), synergistically contribute to neuronal death [132].

Last but not least, the astrocytic SASP can activate microglia and promote neuroinflammation [273].

## 4. Therapeutic Strategies Targeting Neuroinflammation in Alzheimer’s Disease

Because AD is a disease with insidious onset and progressive course, one of the major challenges is to correctly identify patients with AD and to estimate the likelihood of progression in each individual patient [293]. Accumulated evidence shows that for about 15–20 years, AD pathology builds up without clinical evidence of cognitive impairment (preclinical AD). The clinical onset coincides with a relative abrupt increase in tau pathology paralleled by synaptic and neuronal loss, while amyloid burden even decreases with the clinical progression of the disease. Microglial and astrocytic activation show a rather steady progression [153]. The clinical symptoms reported in patients with mild cognitive impairment (MCI) and AD are presented in Table 2.

The scientific community has struggled to identify both clinical tests [294], as well as reliable biomarkers, able to identify in vivo the presence of AD pathology. MRI is able to show medial temporal lobe atrophy, while 18fluorodeoxyglucose (18FDG)-PET can identify posterior cingulate and temporoparietal hypometabolism. Identifying cortical amyloid β deposition on amyloid-PET imaging was proven to improve diagnostic accuracy by the ABIDE and IDEAS studies [158,295]. Tau-PET ligands allow for the identification of the neurofibrillary tangle pathology, which correlates better with cognitive impairment, as well as with the progression of the disease [296], while PET ligands targeting SV2A can explore brain synaptic density [297].

Fluid biomarkers can be measured both in the cerebrospinal fluid and serum of patients. Amyloid β42 and β40, total tau, and phosphorylated tau 181 are validated markers [298], while novel biomarkers are currently evaluated. Neurogranin may reflect synaptic dysfunction [299], while microglia and astrocyte biomarkers could help to monitor the treatment effect [164]. The detection of the soluble fragment of TREM2 in the CSF may be a marker for transition from preclinical to clinical AD [178]. In the blood, phosphorylated tau 181 and 217 were recently shown to reliably differentiate AD from other dementias [300,301].

As a consequence, the proposed diagnostic criteria have undergone considerable changes over time, as shown in Table 3.

However, not every individual with biomarkers for AD will progress to clinically overt AD, which is why the International Working Group for New Research Criteria for the Diagnosis of AD (IWG) recommends against AD biomarker assessment in cognitively unimpaired individuals [306]. Identifying patients at risk for disease progression and stratifying the risk, either with clinical markers or with biomarkers, becomes imperative both for including these patients in clinical trials for the evaluation of disease-modifying agents and for prescribing more aggressive therapy, such as the recently approved aducanumab [307]. A possibility would be to identify specific microRNAs (miRNAs), shown to be altered in neuroinflammatory disorders and in the plasma or CSF of patients, thereby allowing for the early identification of the disease and the initiation of treatment. Research has shown alterations in a series of mRNAs, such as miRNA-155 (pro-inflammatory mediator), miRNA-146a (a negative regulator of inflammation), miRNA-124 (a brain-specific anti-inflammatory miRNA) in the CSF and of miRNA-21 (anti-inflammatory regulator) and let-7 (which promote M2 polarization of macrophages) in the plasma of patients with neurodegenerative disorders [308]. Unfortunately, the complex neurobiology of AD makes the stratification of patients difficult and challenging for physicians.

### 4.1. Early Attempts of Anti-Inflammatory Treatment in AD

Epidemiological studies have suggested that the long-term use of non-steroidal anti-inflammatory drugs (NSAIDs) was linked with the decreased risk of AD [309], a finding reinforced by demonstrating the positive effects of NSAIDs in animal models [310]. However, when evaluated in clinical trials, NSAIDs failed at showing benefits, except for a small study using indomethacin, not subsequently replicated [311], and a follow-up analysis from the ADAPT research group using naproxen [312]. Thus, it appears that in already symptomatic patients, NSAIDs cannot stop the progression of the pathogenic cascades. Nonetheless, a novel non-steroidal anti-inflammatory drug (CHF5074 or itanapraced) lacking cyclooxygenase inhibitory activity was shown to restore normal microglial function, increase phagocytosis, and decrease the production of pro-inflammatory cytokines [313]. The molecule has completed several phase 2 clinical trials (NCT01303744, NCT01602393, and NCT01421056) [314].

However, more specifically targeted anti-inflammatory strategies may be more rewarding.

### 4.2. Anti-Inflammatory Molecules in Clinical Trials

#### 4.2.1. Peroxisome Proliferator-Activated Receptor (PPAR)-γ Agonists

PPAR-γ agonists have been shown to reduce the production of pro-inflammatory cytokines as well as amyloid accumulation in AD mouse models [315]. By activating the ERK pathway, rosiglitazone enhanced cognitive performances in AD models [316] and improved memory in subjects with mild cognitive impairment or early AD, also delaying the decrease in plasma Aβ levels in a small preliminary placebo-controlled study with 4 mg of rosiglitazone for 6 months conducted in 30 subjects (20 with mild AD and 10 controls) [317]. Unfortunately, the results were not replicated in two phase 3, double-blind, placebo-controlled studies (REFLECT-2 and REFLECT-3, NCT00348309 and NCT00348140) conducted on 1496 (REFLECT-2) and 1468 participants (REFLECT-3) randomly assigned to 2 or 8 mg of rosiglitazone for 54 weeks as adjunctive therapy to acetylcholinesterase inhibitors [318]. Pioglitazone reduced the risk for dementia by 47% over a 5-year period in patients with diabetes mellitus, as reported by a large prospective cohort study on 145,928 subjects aged ≥ 60 years and with normal cognition at baseline [319], but a subsequent phase 3 study (NCT01931566, TOMORROW) failed to confirm these initial findings on 3494 participants aged between 65 and 83 years with normal cognition at baseline, randomly assigned to placebo or 0.8 mg pioglitazone daily, with a 5-year follow-up period [320].

#### 4.2.2. Tumor Necrosis Factor-α Inhibitors

Monoclonal antibodies against TNF-α are already in use for autoimmune and inflammatory diseases. Infliximab is a chimeric IgG1 monoclonal antibody that binds to human TNF and which, via intracerebroventricular delivery in mouse AD models, decreases TNF levels, hyperphosphorylated tau, and Aβ plaque burden [321]. Etanercept is a combination of the Fc portion of human IgG1 with the extracellular domain of TNF receptor 2. A case report drew attention upon a significant cognitive improvement after etanercept in an AD patient with rheumatoid arthritis [322]. Subsequent small, open-labeled studies delivered etanercept via perispinal injection (due to the poor BBB crossing of the molecule) and reported cognitive improvement as well [323]. However, a double-blind study (NCT01068353) of 50 mg subcutaneous etanercept weekly over 24 weeks showed the relative safety of the drug (the increased risk of infections is a known side effect of etanercept) but no statistically significant clinical improvement [324].

The selectively soluble TNF inhibitor XPro-1595 inhibits TNF receptors type 1, is able to cross the BBB [325], and has been shown to reduce Aβ plaques, restore long-term potentiation, and prevent synaptic loss in mice [326]. A phase 1 open-label safety and tolerability study (NCT03943264) on 20 participants has just been completed [314], although the results have not been published so far.

#### 4.2.3. Tyrosine Kinase Inhibitors

Masitinib (AB1010), an oral inhibitor of the migration and activity of mast cells, was tested in a phase 2 trial (NCT00976118) in 34 patients with mild-to-moderate AD, assigned to placebo or two doses (3 and 6 mg/kg/day of masitinib orally), continued for 24 weeks. The trial suggested positive effects [327]. This study was followed by a phase 3 trial, recruiting 721 participants (NCT01872598), which was completed in December 2020 but has not published results [314]. The participants were randomized to 5 groups: 2 placebo arms and 3 treatment arms with 2 fixed dose-groups (3 mg/kg/day and 4.5 mg/kg/day of masitinib) and a group which started with 4.5 mg/kg/day and escalated to 6 mg/kg/day after 3 months. The follow-up period extended for 24 weeks. Another phase 3 trial, aiming to include 600 participants with mild to moderate AD, randomized either to placebo or 3 mg/kg/day of masitinib, increased to 4.5 mg/kg/day after 4 weeks and used together with a cholinesterase inhibitor and/or memantine for 24 weeks is planned but is not yet recruiting (NCT05564169) [314].

A related molecule, dasatinib, is currently being evaluated in combination with the antioxidant quercetin in 4 active studies listed on the www.clinicaltrials.gov homepage, accessed on 17 November 2022. A phase 1 open-label trial (NCT04063124, STomP-AD) on 5 participants with AD who will receive 6 consecutive cycles of 100 mg dasatinib and 1000 mg quercetin orally for 2 consecutive days is active but is not recruiting. A phase 1/2 trial is enrolling by invitation up to 20 participants with probable AD to receive the same regimen of dasatinib and quercetin (NCT04785300, ALSENLITE), while 2 studies are currently recruiting patients: a phase 1/2 trial (STAMINA, NCT05422885), aiming to evaluate the effect of 6 cycles of dasatinib 100 mg + 1250 mg quercetin daily for 2 consecutive days on blood-flow regulation and cognition in 12 older adults at risk for AD, and a phase 2 trial (SToMP-AD, NCT04685590) evaluating the efficacy of 6 cycles of 100 mg dasatinib + 1000 mg quercetin daily for 2 consecutive days on cognition in 48 participants with mild cognitive impairment or early-stage AD who are tau PET-positive [314]. The results of these studies are awaited.

#### 4.2.4. MAP Kinase Inhibition

The p38 MAP kinase expressed in glia and neurons mediates the transfer of the γ-phosphate to the hydroxyl group of serine and threonine side chains of substrates, leading to the activation of inflammatory cell signaling cascades and to the enhanced production of IL-1β and TNF-α by microglia in response to stressors, including amyloid-β42. Indeed, brain samples of AD patients showed increased levels of p38 MAPK [328]. Given this association, several selective inhibitors of p38 MAPK have been identified, of which VX-745 (neflamapimod) and MW150 have reached the stage of clinical trials [329,330].

Three phase 2 trials with neflamapimod have been completed (NCT02423200, NCT02423122, and NCT03402659-REVERSE-SD). Trial NCT03435861, a phase 2 study looking specifically at inflammatory biomarkers, was launched in October 2018 and is listed as currently recruiting [314]. Although the oral molecule had significant effects on inflammatory biomarkers, the clinical benefit of the treatment extending over 24 weeks was negligible [331]. Nonetheless, according to the sponsor, in Lewy-body dementia, 120 mg neflamapimod orally/day had improved effects on cognitive performance compared to 80 mg/day. The development of drugs targeting this mechanism is continuing with the synthesis of skepinone derivatives [332].

#### 4.2.5. Other Anti-Inflammatory Strategies

NE 3107, an insulin-sensitizing, orally bioavailable small molecule that binds to ERK and inhibits ERK- and NF-κB-stimulated inflammatory pathways [333] has completed a phase 2 trial (NCT05227820) and is currently evaluated in a phase 3 trial (NCT04669028) aiming to include 316 participants that will last until January 2023 [314].

TB006, a monoclonal antibody targeting galectin-3, a protein binding to Aβ and acting as glue in promoting Aβ oligomerization, has been evaluated in a phase 1/2 trial (NCT05074498), and safety has been assessed even long-term in NCT05476783. Currently, a dose-escalation phase 1 study (NCT04920786) is recruiting patients [314].

Baricitinib, an oral Janus kinase inhibitor approved for treatment in rheumatoid arthritis [334], was shown to reduce inflammatory biomarkers and neural cell death in a human neural cell culture model of inflammatory-mediated death in a dose-dependent manner and was identified by computational biology studies of gene expression profiles of AD brains termed DRIAD (drug repurposing in AD) as one of the leading drugs that reversed the impaired inflammatory signaling in AD [335]. It is being currently evaluated in a phase 1 trial (NCT05189106, Neurodegenerative Alzheimer’s Disease and Amyotrophic Lateral Sclerosis (NADALS) Basket Trial) to assess the safety and BBB penetrance of the molecule.

Several monoclonal antibodies targeting various receptors may also interrupt several pathogenic neuroinflammatory cascades. AL002 targets TREM2 receptors and is being evaluated in a phase 2 trial recruiting 265 participants (NCT04592874, INVOKE-2). AL003 is directed against CD33, expressed exclusively by microglia and macrophages in the brain and identified by GWAS to be among the leading risk factors for AD [186]. In CD33 knockout mice, amyloid load was reduced in the brain and phagocytic clearance of Aβ was enhanced [336]. The safety and tolerability of AL003 have been investigated in a phase 1 trial (NCT03822208), but no results have been published [314]. Daratumumab, a humanized IgG1κ monoclonal antibody that targets the CD38 epitope, has been approved for refractory cases of multiple myeloma. CD38 is a NAD glycohydrolase expressed by microglia, astrocytes, and neurons, shown to have important roles both in neuroinflammation and neural repair processes [92]. Daratumumab is currently tested in a phase 2 trial (NCT04070378, DARZAD) on 15 participants and estimated to be completed in June 2023 [261]. Unfortunately, it may trigger antibody-dependent cell-mediated cytotoxicity (as does isatuximab) [337]. Other small molecules inhibiting CD38 have either poor capability of crossing the BBB or have an IC_50_ in the micromolar range [92]. Mediators of neuroinflammation, such as IL-1β, can also be targeted by monoclonal antibodies, such as canakinumab, assessed in a phase 2 study on 90 participants (NCT04795466). Edicotinib (JNJ-40346527) attenuates microglial proliferation by antagonizing colony-stimulating factor 1 receptor. A phase 1 trial is currently recruiting 54 participants to assess safety and tolerability (NCT04121208), while sargramostim, a granulocyte macrophage colony-stimulating factor with anti-apoptotic and neurogenesis-promoting effects [338], is being evaluated in a phase 2 trial (NCT04902703, SESAD).

A derivative of thalidomide, lenalidomide, is a novel immunomodulatory drug used in myelodysplastic syndromes, which acts by modulating the substrate specificity of the CRL4^CRBN^ E3 ubiquitin ligase. It is currently being evaluated in a phase 2 trial in mild cognitive impairment (NCT04032626, MCLENA-1).

Semaphorin 4D play a key role in regulating the transition between homeostatic and reactive microglia and activating NF-κB [339]. Pepinemab, a monoclonal antibody against SEMA4D, already used in certain forms of cancer, is being tested in a phase 1/2 trial (NCT04381468, SIGNAL-AD) on 40 participants.

Regulatory T cells can be used to reduce neuroinflammatory responses. Research has implicated both the innate immune system and the adaptive immune system in the pathogenesis of AD, showing the increased recruitment of microglia toward the site of amyloid deposition [340], as well as increased CD8^+^ and CD3^+^ cells positively correlated with tau pathology [170,341] in the brain, while in the peripheral blood, patients with AD had decreased regulatory T cells (Tregs) and increased Th17 cells [342,343]. Treg depletion accelerated cognitive decline, while increasing Treg number reversed the cognitive deficits of APP/PS1 mice [344]. Two trials using Tregs isolated from the patients’ blood are currently ongoing. NCT03865017 is a phase 1/2 trial, while NCT05016427 is in phase 1 [314]

Emtricitabine is a repurposed drug, being a nucleoside reverse transcriptase inhibitor used in the treatment of HIV, but which is believed to reduce neuroinflammation. It is currently being evaluated in a phase 1 trial (NCT04500847, LINE-AD) [314].

Table 4 provides an overview of active and recruiting clinical trials with anti-inflammatory molecules, without recording the completed trials or the ones whose status is listed as “unknown” on the clinicaltrials.gov homepage, accessed on 17 November 2022.

### 4.3. Anti-Inflammatory Strategies in Animal Models and In Vitro

Other molecules with anti-inflammatory properties are under research in animal models or in vitro.

#### 4.3.1. Targeting TNF Signaling

TNFR1-specific antibodies, such as ATROSAB, have been shown to shift microglial TNF signaling toward the anti-inflammatory and neuroprotective TNFR2 pathway in a chemical lesion of the nucleus magnocellularis [345]. A similar effect has been obtained by stimulating TNFR2 receptors with specific agonists [346]. One such agonist is the soluble EHD2-scTNFR2, which was tested in combination with ATROSAB in the nucleus basalis magnocellularis chemical lesion model by Dong et al., who showed the efficacy of the combination strategy in treating acute neurodegenerative lesions caused by excitotoxicity [345].

Adalimumab, another TNF-α specific monoclonal antibody used in human patients for peripheral conditions, significantly attenuated neuroinflammation and neuronal damage while also decreasing BACE-1 expression and amyloid load in rodent models of AD [347].

#### 4.3.2. Targeting the cGAS-STING Pathway

The cGAS-STING pathway seems also to be a promising target. Suramin was tested in human monocytic leukemia cells, shown to inhibit the synthesis of 2,3-cGAMP, and downregulate the production of interferon-β [348]. Antimalarial drugs, such as quinacrine hydrochloride, hydroxychloroquine, or 9-amino-6-chloro-2-methoxyacridine have been shown to decrease cGAMP levels in mouse connective tissue cells by interfering with the binding of cGAS to DNA [349]. A competitive inhibitor that binds to the nucleotide binding site of cGAS and inhibits cGAS activity, PF-06928215, was identified in an in vitro assay screen for cGAS inhibitors [350], while the small molecules RU521 and RU365 inhibit the catalytic activity of cGAS in the macrophages of genetically engineered autoimmune mice [351]. In the same transgenic Trx1^-/-^ mice, cGAMP accumulation was reduced by dorsomorphin (compound C) [352]. The palmitoylation of STING leads to the decreased production of pro-inflammatory cytokines and can be achieved with nitro-fatty acids and CXA-10 (10-nitro-9(E)-octadec-9-enoic acid in a variety of cell cultures. CXA-10 is currently assessed in clinical trials for pulmonary arterial hypertension (NCT03449524, NCT04053543) and focal segmental glomerulosclerosis [314,353]. Nitrofuran derivatives (C-178 and C-176) act by blocking the palmitoylation of STING induced by its activation, as shown in bone-marrow-derived macrophages and Trex1^-/-^ mice [354]. Natural chlorinated cyclopentapeptides and astin C attenuated the autoinflammatory response in bone-marrow-derived macrophages from Trx1^-/-^ mice by binding to STING, reducing the affinity of cGAMP to STING, and preventing IRF3 recruitment to STING [355]. Tetradroisoquinolone acetic acids stabilize the inactive conformation of STING and bind to the cGAMP binding site in cell cultures [356]. In neurodegenerative diseases, attempts have been made to attenuate DNA damage and the activation of the cGAS-STING pathway by augmenting NAD+ via the oral administration of nicotinamide riboside in APP/PS1 mice [357] or via NAD+ supplementation [358]. However, given the risk of acute infections or cancer incurred by the prolonged suppression of the neuroinflammatory response, there is currently no inhibitor of the cGAS-STING pathway under preclinical research for AD [359].

Since STING is activated by TBK1, the inhibition of TBK1 could additionally attenuate the inflammation ignited by this pathway. Aminopyrimidines, as inhibitors of both TBK1 and IKK, have been successfully tested in cancer cell lines [360]; GSK8612, developed by GlaxoSmithKline, inhibited IRF-3 phosphorylation and interferon-β secretion in vitro [361], while amlexanox, already approved for use in asthma and aphtous ulcer, is also a dual inhibitor of both IκB kinases and TBK1. Amlexanox exhibits anticancer effects and has potential therapeutic benefits in the treatment of diabetes and obesity [362].

#### 4.3.3. Targeting the Inflammasome

Since the NLRP3 inflammasome is activated by Aβ and contributes significantly to age-related cognitive decline, inhibiting it has gained attention as a potential therapeutic strategy [363]. MCC950 is a potent NLRP3 inhibitor shown in APP/PS1 mice to promote microglial Aβ clearance and improve cognitive function [364]. Together with another NLRP3 inflammasome inhibitor, inzolemid, MCC950 is expected to move into clinical trials for AD as well as Parkinson’s disease and motor neuron disease [34].

#### 4.3.4. Targeting Immune Checkpoints

The programmed cell death-1 (PD-1) receptor expressed on activated T cells together with its ligand (PD-L1) play important roles in maintaining immune homeostasis. Persistent antigen stimulation increases the expression of PD-1 and other immune checkpoint receptors, leading to an increased interaction with ligands on antigen-presenting cells and inducing a hypofunctional state of T cells [365]. The described mechanism can be manipulated to inhibit or enhance the immune response. The antiPD-1/PD-L1 strategy is already used in cancer therapy [366] and immune neutralization of TRAIL (tumor necrosis factor-related apoptosis inducing ligand), which modulates the function of Tregs, has been shown in mouse models of AD to reduce neuroinflammation [367] and improve cognition [368]. However, available antibodies against PD-1 developed for other diseases activated the peripheral immune system but had little effect on macrophage infiltration and the progression of amyloid pathology, indicating the need for further research [369].

#### 4.3.5. Targeting the Complement

A humanized immunoglobulin G4 recombinant antibody against C1q, ANX005, has been proven to be neuroprotective and prevented synaptic loss in a mouse model of AD [166], opening the way for clinical trials [370]. The C3a receptor antagonist SB290157 is able to decrease amyloid load, the Aβ42/40 ratio [371], and microglial proliferation [372], as well as tau hyperphosphorylation [373], while the inhibition of C5a receptor 1 with the cyclic hexapeptide PMX205 decreased amyloid β and tau accumulation, reduced glial activation, and improved cognition in murine AD models [374].

#### 4.3.6. Cell-Based and Cell-Derived Therapeutic Strategies

Extracellular vesicles (EVs) are cell-derived bilayer membrane structures, which carry proteins, lipids, and miRNAs, and mRNAs are involved in the communication between cells and tissues [375] and in regulating cell differentiation, immune response, and tissue repair [55]. Tested in a mouse model of AD, human umbilical cord mesenchymal-stem-cell-derived EVs modulated microglial activation and reduced neuroinflammation and amyloid deposition [376]. In addition, by manipulating the parent cells and incorporating specific microRNAs to target pathways that are impaired in AD, EVs could prove to be a valuable strategy to attenuate the neuroinflammatory response and its consequences [377,378].

Stem cell therapies are also actively being pursued in various neurodegenerative disorders [55,379] and cerebrovascular diseases [380]. Although the use of embryonic stem cells raises a series of ethical issues, mesenchymal stem cells can be harvested from various sources (adipose tissue, bone marrow, liver, tooth buds, cord blood, or placenta) [381], and Takahashi and Yamanaka succeeded in generating induced pluripotent stem cells from somatic cells by using the retroviral transduction of four genes (two transcription factors: the octamer-binding transcription factor 4 and the sex-determining region Y-box, and two signaling factors regulating cellular proliferation and differentiation: the Kruppellike factor 4 and the avian myelocytomatosis viral oncogene homolog, or c-Myc) [382]. While not denying the many issues that still need research regarding the potential tumorigenesis, appropriate number of cells, and convenient delivery methods, stem cells can not only replace lost neurons and glial cells (astrocytes and oligodendrocytes) and be integrated into functional neuronal circuits [380] but also release a series of cytokines and growth factors that can modulate and diminish the neuroinflammatory cascades [380].

#### 4.3.7. Nanotechnology-Based Anti-Inflammatory Approaches in AD

Nanotechnology is an exciting research field with applications in AD both for early diagnosis and treatment [381].

MRI imaging using superparamagnetic iron oxide nanoparticles coated with the fluorescent curcumin or with antibodies against Aβ peptide as a contrast agent allows for the detection of amyloid plaques in vivo. Wrapping the nanoparticle in sialic acid increases sensitivity [383]. Moreover, nanoparticles exposed to biological fluids are covered by a protein corona, and the analysis of the proteins of the corona can offer valuable information on disease stage and severity [384].

Nanoparticles can be used in therapy as well. The organic nanoparticles could improve the delivery of a series of phytochemicals with multitargeted mechanisms of action, such as curcumin, resveratrol, or even other molecules across the BBB, thereby avoiding systemic side effects [385,386], while of the inorganic ones, gold nanoparticles have attracted most attention. They can connect to Aβ and can be used to dissolve the aggregates by delivering thermal energy from a microwave field [387]. They have also been shown to prevent neuroinflammation and cognitive impairment in a rat model of dementia [388]. Exposure to streptozotocin leads to increased levels of IL-1β and NF-κB, serving as a model for sporadic dementia. Gold nanoparticles attenuated neuroinflammation [389], contributed to BBB repair, and also reduced the magnitude of systemic inflammation [390] in hypercholesterolemic rats.

#### 4.3.8. Other Anti-Inflammatory Strategies

Blockade of specific SASP factors, such as IL-6, with available monoclonal antibodies like siltuximab or tocilizumab (anti-human IL-6 receptor) could be a promising approach as well [119].

Calorie restriction has been shown to attenuate the age-related increase of pro-inflammatory cytokines NF-κB, IL-1β, Il-6, or TNF-α and act through the AMPK, mTOR, and Nrf2 pathway to increase lifespan and enhance cognition [391].

Melatonin suppresses pro-inflammatory pathways, NLRP3 activation, cytokine release by SASP, TLR4, and mTOR signaling and shifts microglia towards an M2 anti-inflammatory phenotype [25].

Aside from acting as antioxidants, a series of phytochemicals, such as resveratrol or curcumin, also exhibit anti-inflammatory properties [392], although their ability to cross the BBB is poor [393]. Nanoparticle-mediated delivery could help overcome this drawback [55].

Finally, normalizing the gut microbiota through supplementation with specific strains of *Lactobacilli* and *Bifidobacteria* attenuates inflammaging by downregulating interferon-γ and TNF-α and upregulating Il-10 [394], thus being a non-invasive means to decrease neuroinflammation via the gut–brain axis [395,396].

## 5. Conclusions

Although AD is a complex and heterogenous disease with multiple factors such as age, genetic factors, obesity, hypercholesterolemia, diabetes, or gut dysbiosis contributing to its development and progression, compelling evidence implicates inflammation and neuroinflammation in the conversion of age-related cognitive decline to dementia. Therefore, anti-inflammatory compounds could be a useful tool in delaying the onset and slowing the progression of AD.

The stratification of risk and personalized treatment approaches probably play a crucial role in the success of anti-inflammatory strategies. They may delay the onset of cognitive decline in persons at risk for AD due to comorbidities shown to contribute to inflammaging, such as diabetes, obesity, or hypertension, while in later stages, they could be used as add-on strategies together with the already approved treatments (which offer only limited benefit) or with Aβ- or tau-targeted therapies.

However, much research is still required to identify the best anti-inflammatory strategies and the proper moment for their use. The more potent a drug is, the more serious its side effects, and a careful balancing of risks versus benefits is needed to avoid causing serious and life-threatening infections or tumorigenesis [359]. Modulating neuroinflammation via miRNAs delivered via extracellular vesicles is appealing, but the research is still in its infancy, as is genetic editing via antisense oligonucleotides or CRISPR/Cas9 technology [379] which could inhibit the expression of genes shown to cause familial cases of AD.

## Figures and Tables

**Table 1:** Effects of cytokines and growth factors in the AD brain.

Mediator	Functions	References
Protective effects		
IL-1β	Increases α- and γ-secretases, downregulates BACE-1, promotes Aβ clearance	[196,197]
IL-1α	Increases α-secretase, increases sAPPα, decreases amyloidogenic APP processing	[198]
CXCL10	Decreases Aβ deposition	[199]
CX3CL1	Decreases Aβ deposition, upregulates phosphorylated tau	[200]
Brain derived neurotrophic factor (BDNF)	Promotes the non-amyloidogenic pathway, upregulates sAPPα, dephosphorylates tau via TrkB and PI3K signaling, improves memory performance	[201]
Glial derived neurotrophic factor (GDNF)	neuroprotection	[202]
Nerve growth factor (NGF) Neurotrophin 3 Neurotrophin 4	Modulates microglial polarization toward a non-inflammatory phenotype Limits cleavage of caspases 3,8, and 9, upregulates neuronal apoptosis inhibitory protein Regulates tau dephosphorylation	[203] [204,205] [206]
Enhance AD pathology		
IL-4	Upregulates Aβ production, increases tau phosphorylation	[207]
IL-6	Increases tau phosphorylation, increases amyloid plaque burden	[208]
IL-8	Increases tau phosphorylation, promotes Aβ deposition	[209]
IL-10	Promotes Aβ deposition	[210]
IL-18	Upregulates BACE-1 and γ-secretase, enhances Aβ formation	[211]
TNF-α	Upregulates BACE-1 and γ-secretase, increases sAPPβ	[212]
Transforming growth factor (TGF)-β	Increases Aβ deposition	[213]
CCL2	Enhances amyloid production and deposition, accelerate tau pathology	[214]
CCL3	Promotes infiltration with T lymphocytes, upregulates BACE-1	[215,216]
CCL5	Promotes T cell infiltration in the brain, enhances microglial proliferation	[217]
Interferon-γ	Upregulates BACE-1 and γ-secretases, increases amyloid accumulation	[218]

**Table 2:** Clinical picture in the different stages of AD (adapted form Ogunmokun et al. [206]).

Stages	Clinical Picture
Early onset AD/MCI	Difficulty in word-finding, impairment in reasoning and judgement
Mild AD	Memory loss, misplacing items, restlessness, anxiety, altered personality, episodes of aggression
Moderate AD	Attention deficit, recognition problems, confusion, delusions, paranoia, hallucinations, impulsive behavior
Severe AD	Severe dementia, functional limitations, swallowing difficulties, loss of bladder/bowel control, weight loss, seizures, enhanced diurnal sleep time with nocturnal insomnia

**Table 3:** Proposed diagnostic criteria for AD.

	NINCDS-ADRDA Criteria, 1984 [302]	NIA-AA, 2011 [303]	IWG-AA, 2016 [304]	NIA-AA, 2018 [305]	IWG, 2021 [306]
Setting	Research and clinical	Research and clinical	Research	Research	Research and clinical
Clinical requirements	Memory changes and other cognitive impairments	Amnestic or non-amnestic mild cognitive impairment, or dementia	None	None	Amnestic syndrome of hippocampal type, primary progressive aphasia of the semantic, non-fluent, or logopenic variant, corticobasal syndrome, behavioral or dysexecutive frontal syndromes
Biological requirements	None	Amyloid β marker (CSF or PET) or marker of degeneration (CSF tau, phosphorylated tau, ^18^F-fluorodeoxyglucose-PET, and T1-weighted MRI)	Amyloid β marker (CSF or PET) and tau marker (CSF or PET)	Amyloid β marker (CSF or PET) and tau marker (CSF or PET)	Amyloid β marker (CSF or PET) and tau marker (CSF or PET)

ADRDA = Alzheimer’s Disease and Related Disorders Association (now the Alzheimer’s Association) Work Group. IWG = International Working Group criteria. IWG–AA = International Working Group and Alzheimer’s Association joint criteria. NIA–AA = US National Institute on Aging and Alzheimer’s Association joint criteria. NINCDS = US National Institute of Neurological and Communicative Disorders and Stroke criteria.

**Table 4:** Active and recruiting clinical trials evaluating anti-inflammatory strategies (as listed on www.clinicaltrials.gov [314]).

Phase, Status	Molecule	Trial Identifier	Number of Participants	Estimated Date of Completion	Sponsor
Not applicable, recruiting	AL002	NCT03671880	30	December 2024	InSightec
Phase 1, recruiting	TB006	NCT04920786 (TB006SAD)	48	January 2023	TrueBinding, Inc.
Phase 1, recruiting	VT301 (regulatory T cells)	NCT05016427	12	April 2022	VTBIO Co., LTD
Phase 1, recruiting	emtricitabine	NCT04500847 (LINE-AD)	35	August 2023	Butler Hospital, The Miriam Hospital, Alzheimer’s Association, Brown University
Phase 1/2, active, not recruiting	Dasatinib + quercetin	NCT04063124	5	December 2023	University of Texas Health Science center at San Antonio, Mayo Clinic
Phase 1/2, enrolling by invitation	Dasatinib + quercetin	NCT04785300, ALSENLITE	20	December 2023	Mayo Clinic
Phase 1/2, recruiting	Dasatinib + quercetin	NCT05422885 STAMINA	12	June 2023	Hebrew Senior Life
Phase 1/2, recruiting	TB006	NCT05074498	140	October 2022	TrueBinding, Inc.
Phase 1/2, recruiting	Baricitinib	NCT05189106 (NADALS)	265	January 2024	Alector Inc; AbbVie
Phase 1/2, recruiting	Pepinemab	NCT04381468 SIGNAL-AD	40	February 2024	Vaccinex Inc; Alzheimer’s Drug Discovery Foundation; Alzheimer’s Association
Phase 2, recruiting, open-label	XPro-1595	NCT05522387	261	December 2025	Immune Bio, Inc.
Phase 2, not yet recruiting	XPro-1595	NCT05321498	60	January 2023	Immune Bio, Inc.
Phase 2, recruiting	XPro-1595	NCT05318976 MINDFuL	201	June 2023	Immune Bio, Inc.
Phase 2, recruiting	Dasatinib + quercetin	NCT04685590SToMP-AD	48	January 2023	University of Texas, Wake Forest health Sciences
Phase 2, recruiting	VX-745, Neflamapimod	NCT03435861	40	June 2021	University Hospital of Toulouse
Phase 2, long term extension study, active, not recruiting	TB006	NCT05476783	180	October 2024	TrueBinding, Inc.
Phase 2, recruiting	AL002	NCT04592874INVOKE-2	265	January 2024	Alector Inc; AbbVie
Phase 2, recruiting	Daratumumab	NCT04070378 DARZAD	15	June 2023	Janssen Scientific Affairs, LLC
Phase 2, recruiting	Canakinumab	NCT04795466	90	February 2026	Novartis Pharmaceuticals
Phase 2, recruiting	Sargramostim	NCT04902703 SESAD	42	July 2024	University of Colorado, National Institute of Aging; Alzheimer’s Association; Partner Therapeutics, Inc.
Phase 2, recruiting	Lenalidomide	NCT04032626 MCLENA-1	30	September 2024	St Joseph’s Hospital and Medical Center Phoenix; National Institute of Aging; The Cleveland Clinic
Phase 3, recruiting	NE3107	NCT04669028	316	January 2023	BioVie, Inc.
Phase 3, not yet recruiting	Masitinib	NCT05564169	600	December 2025	AB Science

## Abbreviations

AD	Alzheimer’s disease
ADAM	A disintegrin and metalloproteinase
AIM2	absent in melanoma 2
Akt	protein kinase B
AMP	adenosine monophosphate
AMPK	5′-adenosine monophosphate (AMP)-activated protein kinase
AP1	activator protein 1
APOE	apolipoprotein E
APP	amyloid precursor protein
ARE	antioxidant response element
ASC	apoptosis-associated speck-like protein containing CARD
ATG	autophagy-related protein
ATP	adenosine triphosphate
BACE	β-site APP cleaving enzyme
BBB	blood brain barrier
BDNF	brain-derived neurotrophic factor
BER	base excision repair
CAMKII	calcium/calmodulin-dependent protein kinase II
CARD	caspase activation and recruitment domain
CAT	catalase
CBP	CREB binding protein
CCL	C-C motif chemokine ligand
CGAS	cyclic GMP-AMP synthase
CIP/KIP	CDK interacting protein/kinase inhibitory protein
CNS	central nervous system
COX	cyclooxygenase
CRISPR/Cas9	clustered regularly interspaced short palindromic repeats/CRISPR-associated protein 9
CSF	cerebrospinal fluid
CXCL-CXCL	C-X-C motif ligand
CSF1R	colony stimulating factor 1 receptor
DAMPS	damage-associated molecular patterns
DAP12	DNAX-activation protein of 12 kDa
DNA	deoxyribonucleic acid
Drp1	dynamin-related protein 1
EAAT	excitatory amino acid transporter
EMRE	essential MCU regulator
ER	endoplasmic reticulum
ERK	extracellular-regulated kinase
ETC	electron transport chain
EVs	extracellular vesicles
FGF21	fibroblast growth factor 21
FIP200	focal adhesion kinase (FAK) family-interacting protein of 200 kDa
Fis1	mitochondrial fission 1 protein
GABA	γ-aminobutyric acid
GDF15	growth/differentiation factor 15
GDNF	glial-derived neurotrophic factor
GFAP	glial fibrillary acidic protein
GMP	guanosine monophosphate
GPx	glutathione peroxidase
GSH	glutathione
GSK3β	glycogen synthase kinase-3 beta
GWAS	genome-wide association studies
HIF	hypoxia-inducible factor
HIV	human immunodeficiency virus
HMGB	high mobility box group
HMGCR	3-hydroxy-3-methylglutaryl CoA reductase
HO-1	heme oxygenase-1
IC_50_	half maximal inhibitory concentration
ICAM1	intercellular adhesion molecule 1
IF	interferon
IFNAR	interferon-α/β receptor
IκB	inhibitor of κB
IKK	IκB kinase
IL	interleukin
IMM	inner mitochondrial membrane
IP3Rs	inositol trisphosphate receptors
IRF	interferon regulatory factor
ISGF3	interferon-stimulated gene factor-3
JAK	Janus kinase
JIP	c-Jun N-terminal kinase-interacting protein
JNK pathway	Jun N-terminal kinase pathway
Keap1	kelch like erythroid cell-derived protein with CNC homology (ECH)-associated protein 1
LAMP1	lysosomal-associated membrane protein 1
LC3	light chain 3
MAMs	mitochondria-associated membranes
MAPK	mitogen-activated protein kinase
MCI	mild cognitive impairment
MCU	mitochondrial membrane Ca^2+^ uniporter
MCUR1	MCU regulator 1
Mfn	mitofusin
MHC	major histocompatibility complex
MICU 1 and 2	mitochondrial calcium uptake 1 and 2
miRNA	microRNA
MMP	matrix metalloproteinase
MPTP	mitochondrial permeability transition pore
MRI	magnetic resonance imaging
mRNA	messenger RNA
mTOR	mammalian target of rapamycin
mtDNA	mitochondrial DNA
NAD	nicotinamide adenine dinucleotide
NADH	reduced nicotinamide adenine dinucleotide
NCLX	Li^+^-permeable Na^+^/Ca^2+^ exchanger
NGF	nerve growth factor
NFATs	nuclear factor of activated T cells receptors
NF-κB	nuclear factor-kappa B
NFT	neurofibrillary tangle
NLR	nucleotide-binding oligomerization domain (NOD)-like receptor
NO	nitric oxide
NOS	nitric oxide synthase
NOX	NADPH oxidase
Nrf2	nuclear factor erythroid 2-related factor 2
NSAIDs	non-steroidal anti-inflammatory drugs
NSF	N-ethylmaleimide sensitive fusion protein
OH8dG	nucleoside 8-hydroxy-2′-deoxyguanosine
OMM	outer mitochondrial membrane
OPA1	optic atrophy 1
OXPHOS	oxidative phosphorylation
PAMPs	pathogen-associated molecular patterns
PARP	poly(ADP-ribose) polymerase
PET	positron emission tomography
PGAM	phosphoglycerate mutase
PGC-1α	peroxisome proliferator-activated receptor gamma coactivator 1-alpha
PI3K	phosphatidylinositol 3-kinase
PINK1	PTEN-induced kinase 1
POLG	DNA polymerase subunit gamma
PQBP1	polyglutamine binding protein 1
PRR	pattern recognition receptor
PSEN	presenilin
RAGE	receptor for advanced glycation endproducts
ROS	reactive oxygen species
RUNX1	runt-related transcription factor 1
RyR	ryanodine receptor
SASP	senescence-associated secretory phenotype
SNAREs	soluble N-ethylmaleimide sensitive factor attachment protein receptors
SOD	superoxide dismutase
SR-A	scavenger receptor class A
SR-B1	scavenger receptor B1
SREBP2	sterol regulatory element-binding protein 2
STAT	signal transducer and activator of transcription
STING	stimulator of interferon genes
Syk	spleen tyrosine kinase
TBK1	TANK binding kinase 1
TFAM	mitochondrial transcription factor A
Th	T helper lymphocytes
TIMP1	tissue inhibitor of metalloproteinases 1
TLR	toll-like receptor
TNF	tumor necrosis factor
TNFR	TNF-α receptor
TOM	translocase of the outer membrane
TRAIL	tumor necrosis factor-related apoptosis inducing ligand
Treg	regulatory T cells
TREM	triggering receptor expressed on myeloid cells
TREX1	three prime repair exonuclease 1
ULK	Unc-51-like kinase
VDAC	voltage-dependent anion channel
VGCC	voltage-gated calcium channel
VPS	vascular protein sorting
WIPI	tryptophan-aspartic acid (WD) repeat domain phosphoinositide-interacting proteins
